# NDUFS1‐Mediated Mitochondrial Complex I Activity Maintains Pancreatic Cancer Stemness by Promoting PAX2 Hypomethylation

**DOI:** 10.1002/mco2.70678

**Published:** 2026-03-18

**Authors:** Xin‐Yu Fan, Wen Li, Ying Shi, Bao‐Qing Xu, Hao Wang, Ruo‐Fei Tian, Zi‐Chuan Duan, Jing Fan, Jia‐Rong Liu, Xiu‐Xuan Sun, Bin Wang, Li‐Juan Wang, Ke Wang, Shi‐Jie Wang, Xiang‐Min Yang, Hong‐Yong Cui, Zhi‐Nan Chen, Ling Li

**Affiliations:** ^1^ Department of Cell Biology National Translational Science Center for Molecular Medicine State Key Laboratory of Holistic Integrative Management of Gastrointestinal Cancer State Key Laboratory of New Targets Discovery and Drug Development for Major Diseases Fourth Military Medical University Xi'an China; ^2^ The 900th Hospital of Joint Logistics Support Force Fuzhou China

**Keywords:** CD147, complex I, NDUFS1, pancreatic cancer stem cells, PAX2

## Abstract

Pancreatic cancer is highly refractory and aggressive, with cancer stem cells (CSCs) being primarily responsible for its metastasis and chemoresistance. Deregulated cellular bioenergetics is a hallmark of cancer cells. However, the influence of bioenergetics on the maintenance of pancreatic CSC stemness and its underlying mechanisms have not been fully elucidated. In this study, pancreatic CSCs, isolated either by sorting ALDH^+^ subpopulation or enriching serially passaged tumorspheres from pancreatic cancer cells and PDX model, exhibited active mitochondrial complex I activity and increased oxidative phosphorylation. Complex I maintains stemness and tumorigenicity through its core subunit, NDUFS1. NDUFS1‐mediated pancreatic CSC stemness is reinforced by high expression of CD147, which promotes pSTAT3^Tyr705^‐mediated *NDUFS1* transcription. To promote stemness, CD147‐NDUFS1 initiates SIRT1‐DNMT1 metaboloepigenetic signaling, decreasing promoter hypomethylation and increasing the mRNA expression of the stem cell transcript factor *PAX2*. Moreover, *NDUFS1* and *CD147* expressions were highly correlated in pancreatic cancer tissues, and their co‐expression was significantly associated with poor patient survival. Taken together, our study provides evidence that mitochondrial complex I functions as a key player in CSC stemness maintenance through NDUFS1‐mediated retrograde metaboloepigenetic signaling. Blocking a key regulator of mitonuclear communication by targeting CD147 may be a novel therapy for pancreatic cancer.

## Graphical abstract

Pancreatic cancer stem cells (CSCs) maintain stemness and tumorigenicity through NDUFS1‐mediated complex I activity, which is transcriptionally upregulated via the CD147‐STAT3 signaling axis. Enhanced oxidative phosphorylation (OXPHOS) and NAD+ production driven by this CD147‐NDUFS1 axis subsequently trigger the SIRT1‐DNMT1 metaboloepigenetic signaling cascade, resulting in PAX2 promoter hypomethylation, increased PAX2 expression, and ultimately reinforcement of the CSC stemness.

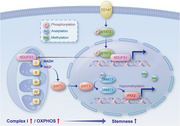

## 1 Introduction

Pancreatic cancer is an aggressive malignant disease with a 5‐year survival rate of <10% [1, 2]. A possible explanation for this poor prognosis is the presence of a small number of cancer stem cells (CSCs) [3]. Therefore, exploring the maintenance of stemness potential in pancreatic CSCs may facilitate the development of new methods for selectively targeting these cells and the design of new and effective therapies for pancreatic cancer.

Deregulated cellular energetics is a hallmark of cancer cells [4, 5, 6]. Although most cancer cells rely on aerobic glycolysis (the Warburg effect) to sustain rapid proliferation [7], pancreatic CSCs exhibit distinct metabolic dependencies. Unlike bulk tumor cells, they primarily depend on oxidative phosphorylation (OXPHOS) for survival, self‐renewal, stemness maintenance, and tumorigenic potential [8, 9]. However, studies have suggested a possible link between glycolysis and stemness in pancreatic ductal adenocarcinoma (PDAC) [10]. Moreover, pancreatic CSCs demonstrate metabolic flexibility during organotropic metastasis, bifurcating into two distinct OXPHOS subtypes: (1) MDR1^+^ drug‐resistant CSCs, which exhibit aerobic glycolysis combined with fatty acid β‐oxidation‐mediated oxidative (glyco‐oxidative) metabolism and metastasize to the liver; and (2) ALDH^+^CD133^+^ CSCs, which predominantly rely on OXPHOS and metastasize to the lung [11]. This context‐dependent metabolic plasticity underscores the need to elucidate the relationship between metabolic phenotypes and pancreatic CSC stemness, which is pivotal for understanding tumor progression and developing effective therapeutic interventions [12, 13, 14, 15].

Emerging evidence suggests that energy metabolism is not merely a hallmark of the CSC phenotype but also a key regulator of intracellular processes, particularly in modulating stemness gene expression and determining CSC fate [16]. Therefore, elucidating how OXPHOS sustains CSC stemness may reveal novel therapeutic targets for eliminating pancreatic CSC. Studies indicate that metabolites generated from OXPHOS or its upstream tricarboxylic acid (TCA) cycle, such as NAD^+^, acetyl‐CoA, succinate, and α‐ketoglutarate, modulate the epigenetic landscape by serving as cofactors for chromatin‐modifying enzymes, thereby orchestrating gene expression programs that govern self‐renewal and stemness maintenance [16, 17, 18, 19, 20, 21]. However, whether OXPHOS directly influences CSC fate through its driving machinery, the electron transport chain (ETC) respiratory complexes remain unclear.

Among the four mitochondrial respiratory complexes, Complex I, the primary entry point for electrons into the ETC, has recently been implicated in sustaining neuroinflammation [22] and promoting kidney cancer metastasis [23]. Notably, pharmacological inhibition of Complex I has emerged as a promising strategy for suppressing OXPHOS in CSCs. For example, metformin, a widely used Complex I inhibitor, induces mitochondrial dysfunction in pancreatic CSCs [9], while rotenone, another potent complex I inhibitor, reduces ATP production in breast CSCs. Given that OXPHOS can influence CSC fate, it is plausible that Complex I directly contributes to the maintenance of CSC stemness. However, the precise role of mitochondrial complexes in determining CSC fate and stemness remains elusive. To date, the function of Complex I in modulating CSC stemness has not been investigated.

This study aimed to systematically characterize the metabolic profiles of pancreatic CSCs and investigate how these metabolic features regulate stemness potential. Our findings demonstrate that Complex I‐derived OXPHOS is a critical metabolic node for sustaining pancreatic CSC stemness. Mechanistically, Complex I initiates a novel mechanism of stemness maintenance that involves the NDUFS1‐mediated metaboloepigenetic signaling cascade. This discovery not only advances our understanding of pancreatic CSC biology but also provides a compelling rationale for developing novel therapeutic strategies targeting metabolic vulnerabilities in pancreatic CSCs.

## 2 Results

### 2.1 Mitochondrial Complex I Activity Maintains Pancreatic CSC Stemness

**FIGURE 1:**
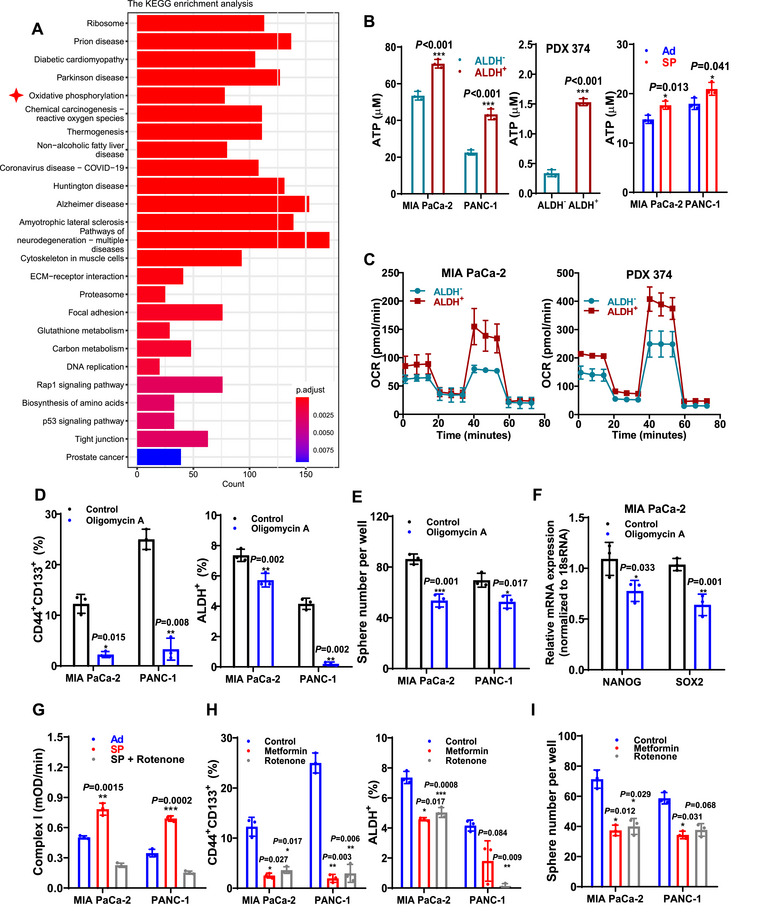
Mitochondrial complex I activity sustains pancreatic CSC stemness. (A) KEGG enrichment analysis of DEGs between patients with high‐ or low‐mRNAsi scores. (B) ATP production in CSCs and non‐CSCs obtained from ALDH^+^ or ALDH^−^ subpopulations (*left* and *middle*) and from passaged tumorsphere (SP) or adherent cell (Ad, *right*). (C) Oxygen consumption rates in CSCs and non‐CSCs from the ALDH^+^ and ALDH^−^ subpopulations. (D–F) CD44+CD133^+^ and ALDH^+^ subpopulation analysis (D), tumorsphere number (E), and *NANOG* and *SOX2* mRNA levels (F) in cells treated with or without oligomycin A. The solvent of oligomycin A was used as a negative control. (G) Mitochondrial complex I activity in Ad, SP, and SP with rotenone. (H and I) CD44^+^CD133^+^ and ALDH^+^ subpopulation analysis (H) and tumorsphere numbers (I) in cells treated with or without rotenone. Metformin was used as a positive control. CSC, cancer stem cell; DEGs, differentially expressed genes.

To identify key metabolic signatures sustaining pancreatic CSC stemness, we analyzed RNA‐seq data from patients with PDAC in the Cancer Genome Atlas database. Patients were stratified into high‐ and low‐mRNAsi groups, where mRNAsi scores served as a semi‐quantitative measure of the stemness phenotype with higher scores indicating greater CSC potential [24, 25]. Subsequent Kyoto Encyclopedia of Genes and Genomes (KEGG) and Gene Ontology (GO) enrichment analysis revealed that oxidative phosphorylation, electron transport chain, and NADH dehydrogenase were among the most significantly enriched pathways (Figure 1A, Figure S1A).

We further verified the metabolic traits of pancreatic CSCs and their contribution to stemness. In in vitro models, CSCs were either sorted as an ALDH^+^ subpopulation or enriched as SP derived from pancreatic cancer cells and the PDX model. When compared with non‐CSCs (the ALDH^−^ subpopulation or Ad), CSCs exhibited significantly higher levels of ATP production and glucose uptake but similar levels of lactate production (Figure 1B, Figure S1B, C). In contrast to non‐CSCs, CSCs displayed higher OCRs but similar ECARs (Figure 1C, Figure S1D–F). Moreover, ATP production inhibition via oligomycin A (an inhibitor of mitochondrial ATP synthase) significantly decreased stemness, as detected by the ratios of CD44^+^CD133^+^ and ALDH^+^ CSCs, spherogenesis capacity, and the expression of the stemness genes *NANOG* and *SOX2* (Figure 1D–F). Notably, treatment with a glycolysis inhibitor (2‐DG), a fatty acid oxidation inhibitor (etomoxir [ETO]), or a glutaminase inhibitor (BPTES) did not significantly inhibit the expression of the four stemness genes or tumorsphere formation (Figure S2A, B). However, an OXPHOS inhibitor (UK 5099) exerted the strongest suppressive effects on all the stemness potentials. These results confirmed that OXPHOS plays a primary role in maintaining pancreatic CSC stemness.

Next, we investigated which specific component of the ETC in OXPHOS was responsible for maintaining the stemness potential of pancreatic CSCs by measuring the enzymatic activities of the four ETC complexes. The results showed that only complex I activity was significantly increased in CSCs (Figure 1G, Figure S2C). Moreover, treatment with the complex I inhibitors, rotenone and metformin, significantly decreased CSC stemness (Figure 1H–I, Figure S2D). In contrast, inhibitors of complex II (3‐nitropropionate acid), complex III (antimycin A), and complex IV (NaN3) had no significant effect (Figure S2E, F). These findings suggested that ETC complex I is essential for pancreatic CSC stemness.

### 2.2 Complex I Core Subunit NDUFS1 Maintains Pancreatic CSC Stemness

**FIGURE 2:**
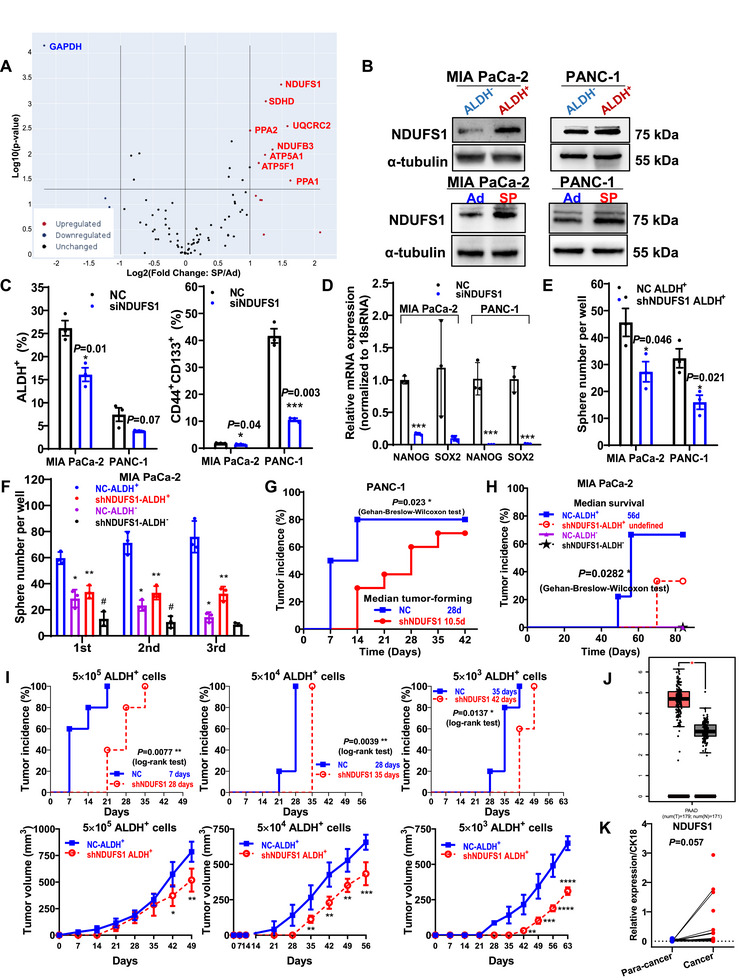
Complex I core subunit NDUFS1 maintains pancreatic CSC stemness. (A) Volcano plot of DEGs between the SP and Ad groups of MIA PaCa‐2 cells analyzed using the Human Mitochondrial Energy Metabolism RT^2^ Profiler PCR Array. (B) Protein levels of *NDUFS1* in ALDH^+^ or ALDH^−^ subpopulations, as well as in SP or Ad cells. (C and D) ALDH^+^ and CD44^+^CD133^+^ subpopulation analysis (C) and *NANOG* and *SOX2* mRNA levels (D) in cells either with or without NDUFS1 interference. (E and F) Tumorsphere numbers in ALDH^+^ or/and ALDH^−^subpopulations, either without (E) or with passage (F). *compared with NC ALDH^+^ subpopulation; #compared with NC ALDH^−^ subpopulation. (G and H) Tumor incidence in nude mice injected with 1 × 10^6^ PANC‐1 cells (G) and in NOD‐SCID mice injected with 50,000 ALDH^+^ or ALDH^−^ MIA PaCa‐2 cells (H), either without or with *NDUFS1* knockdown. (I) Tumor incidence and tumor volume in NOD‐SCID mice injected with 5000–500,000 ALDH^+^ or ALDH^−^ MIA PaCa‐2 subpopulations, either without or with *NDUFS1* knockdown. (J) N*DUFS1* mRNA levels in the GEPIA database. (K) NDUFS1 protein expression in human pancreatic cancer tissues. CSC, cancer stem cell; SP, stem‐like cells; Ad, adherent cell; PCR, polymerase chain reaction.

To identify complex I‐related genes critical for pancreatic CSC stemness, we performed a mitochondrial energy PCR array. The most significantly upregulated gene in SP‐derived CSCs was *NDUFS1* (NADH: ubiquinone oxidoreductase core subunit S1; Figure 2A, Figure S3A). As the largest catalytic subunit of ETC complex I, NDUFS1 was highly expressed in ALDH^+^ and SP‐derived CSCs (Figure 2B, Figure S3B), suggesting its potential role in maintaining CSC properties. Further, *NDUFS1* knockdown significantly decreased the proportion of pancreatic CSCs, stemness‐related gene expression, and tumorsphere‐forming capacity of ALDH^+^ CSCs (Figure 2C–E, Figure S3C–H). Moreover, *NDUFS1* knockdown significantly impaired the self‐renewal ability of ALDH^+^ CSCs, evidenced by their decreased spherogenic capacity over three serial passages (Figure 2F). Notably, this inhibitory effect at the third passage was more pronounced in ALDH^+^ CSCs (57.15 ± 0.07%) than that in ALDH^−^ non‐CSCs (37.06 ± 0.15%), further supporting the critical role of NDUFS1 in CSC maintenance.

Next, to evaluate tumor‐initiating capacity, we employed two complementary models: subcutaneous xenograft models in nude mice using either bulk tumor cells or sorted ALDH^+^ CSCs, and limiting‐dilution assays in NOD‐SCID mice with serially diluted ALDH^+^ CSCs. In both systems, tumor incidence and growth kinetics were monitored as primary endpoints. Notably, *NDUFS1* knockdown in bulk tumor cells significantly attenuated tumorigenic potential, evidenced by reduced tumor incidence, delayed tumor formation, and impaired tumor growth (Figure 2G, Figure S3I). This tumor‐suppressive effect was consistently observed in pancreatic ALDH^+^ CSCs, in which *NDUFS1* knockdown similarly suppressed their tumorigenic capacity (Figure 2H, Figure S3J). Given the negligible tumor‐forming ability of ALDH^−^ non‐CSCs, subsequent experiments focused solely on ALDH^+^ CSCs. Notably, serially diluted ALDH^+^ CSCs with NDUFS1 knockdown exhibited dose‐dependent impairments in tumor initiation, generating significantly fewer and smaller tumors across all inoculated cell doses compared with NC ALDH^+^ CSCs (Figure 2I). These results confirmed that *NDUFS1* is essential for maintaining the stemness and tumor‐initiating capacity of pancreatic CSCs.

To explore the potential clinical and pathological associations between *NDUFS1* and pancreatic cancer, we analyzed *NDUFS1* levels in patient tissues. Data from both public databases and tissue immunofluorescence staining consistently revealed that *NDUFS1* mRNA and protein levels were significantly higher in pancreatic cancer tissues than in adjacent noncancerous pancreatic tissues (Figure 2J–K, Figure S4A). Survival analysis indicated that patients with high *NDUFS1* mRNA expression but no protein expression had shorter median survival times than those with low *NDUFS1* mRNA expression. Moreover, NDUFS1 protein levels significantly correlated with N stage and lymph node invasion (Figure S4B–E, Tables S1 and S2). Collectively, these findings suggest that elevated *NDUFS1* is closely associated with the development of pancreatic cancer and an unfavorable prognosis for patients.

### 2.3 CD147 Promotes Complex I‐Mediated Pancreatic CSC Stemness

**FIGURE 3:**
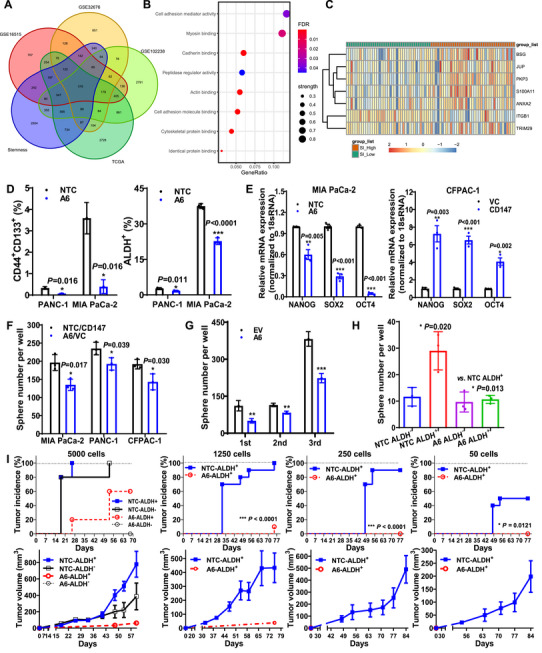
CD147 promotes the stemness and tumorigenicity of pancreatic CSCs. (A) Identification of 316 DEGs selected from the GEO and TCGA databases. (B) Significantly enriched pathways were screened using PPI and enrichment score *p*‐value analyses. (C) Heatmap of seven genes in the cell adhesion mediator activity pathway according to their mRNAsi scores. (D and E) CD44^+^CD133^+^ and ALDH^+^ subpopulation analysis (D) and *NANOG*, *SOX2*, and *OCT4* mRNA levels (E) in *CD147* knockdown (A6) or non‐target control (NTC) cells, as well as in *CD147* knockin (CD147) or control vector (VC) cells. (F–H) Number of tumorspheres in bulk tumor cells under non‐passaged condition (F) or after passage (G), and in 500 cells from either ALDH^+^ or ALDH^−^ subpopulations (H). (I) Tumor incidence (*top*) and tumor volume (*bottom*) in NOD‐SCID mice injected with 50‒5000 cells from the ALDH^+^ or ALDH^−^ subpopulations, with or without *CD147* knock‐down. CSC, cancer stem cell; DEGs, differentially expressed genes; GEO, Gene Expression Omnibus; TCGA, the Cancer Genome Atlas.

To identify the upstream genes that maintain pancreatic CSC stemness by modulating ETC complex I, we screened 316 overlapping differentially expressed genes (DEGs) between patients with high or low mRNAsi expression and those with cancer or normal tissues. Protein‒protein interaction (PPI) network and GO enrichment analyses revealed that the most significantly enriched pathway among these DEGs was the cell adhesion mediator activity pathway. Within this pathway, seven stemness‐related genes were identified, among which *CD147* (*BSG)* was significantly upregulated in patients with high mRNAsi levels (Figure 3A–C).

Building on our earlier findings that CD147 promotes pancreatic cancer development and that its antibody HAb18IgG attenuates pancreatic CSC stemness [26, 27], we further discovered that *CD147* expression (mRNA and protein) was significantly elevated in CSCs compared with non‐CSCs (Figure S5A, B). Strikingly, CD147‐knockdown (A6) not only reduced the proportions of CD44^+^CD133^+^ and ALDH^+^ CSCs but also downregulated core stemness genes (NANOG, SOX2, and OCT4), whereas CD147‐overexpressing (CD147) conversely increased the expression of these core stemness genes (Figure 3D,E, Figure S5C, D). Moreover, CD147 expression profoundly impacted tumor sphere formation, affecting both sphere number and size, with the effect persisting over three passages or even in single‐cell inoculation, indicating a durable impact on self‐renewal capacity (Figures 3F,G, Figure S5E‐G). Critically, *CD147* knockdown reduced, whereas knock‐in increased tumorsphere numbers in ALDH^+^ CSCs and CD44^+^ CSCs (Figure 3H, Figure S5H), confirming the regulatory role of *CD147* in maintaining CSC stemness.

In in vivo experiments, *CD147* knockdown in CSCs significantly inhibited tumor formation and growth. When 5000 cells were inoculated, compared with no‐target control (NTC) ALDH^+^ CSCs, A6 ALDH^+^ CSCs with *CD147* knockdown exhibited a significantly lower tumor incidence, a markedly prolonged median tumor formation time, notably smaller tumor sizes, and a significantly reduced CSC frequency (Figure 3I, Table 1). The inhibitory effects of CD147 knockdown became more prominent as the number of inoculated cells decreased. Remarkably, no tumors were detected in mice inoculated with 250 or 50 A6 ALDH^+^ CSCs. Therapeutic targeting of CD147 with the HcHAb18 antibody markedly reduced tumor incidence and final tumor burden, underscoring its efficacy in targeting CD147‐driven oncogenesis (Figure S5I–K). Consistent with these in vivo findings, patients with pancreatic cancer with high *CD147* expression had significantly shorter median survival times (Figure S5L, Table S2). Both univariate and multivariate Cox proportional hazards analyses revealed that CD147 expression, rather than NDUFS1 expression, was an independent prognostic factor for poor overall survival (Tables S2 and S3). Collectively, these findings strongly suggested that *CD147* plays a crucial role in promoting the stemness and tumorigenicity of pancreatic CSCs.

**TABLE 1:** CD147 promoted in vivo tumor formation ability by enhancing CSC frequency.

Cells per injection	5000	1250	250	50	CSC frequency^a^	*p*‐values
NC‐ALDH^+^	5/5	10/10	9/10	4/10	1/105	
A6‐ALDH^+^	3/5	1/10	0/10	0/10	1/7893	1.96 × 10^−17^
NC‐ALDH^−^	5/5	—	—	—	—	
A6‐ALDH^−^	0/5	—	—	—	—	

^a^ Stem cell frequency was analyzed by extreme limiting dilution analysis (ELDA).

**FIGURE 4:**
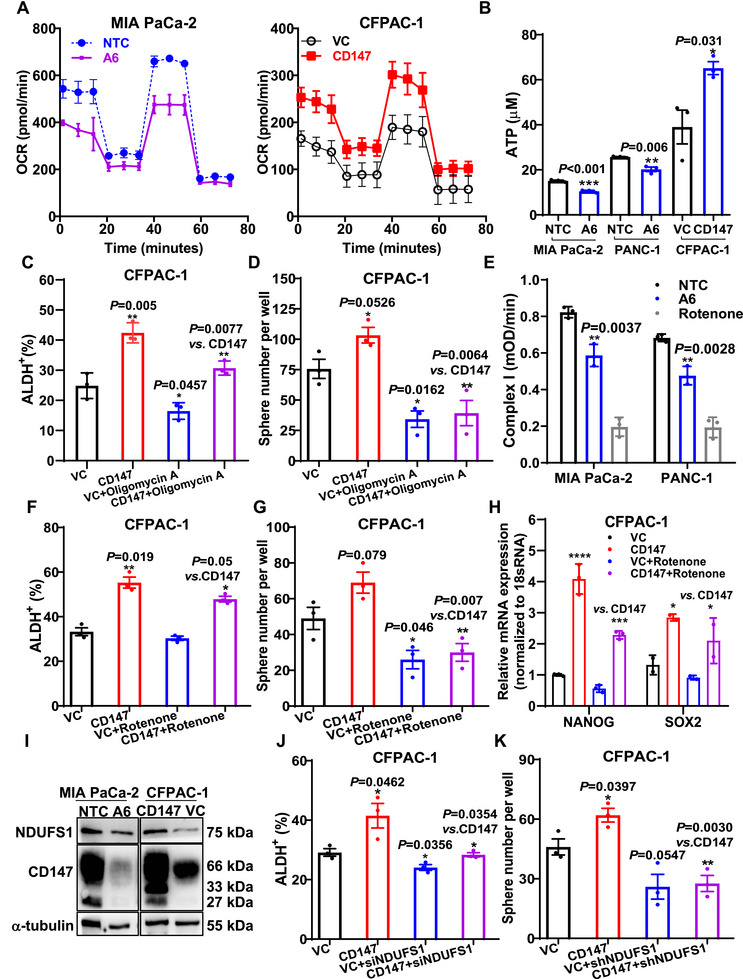
CD147 promotes complex I subunit NDUFS1‐mediated pancreatic CSC stemness. (A) Oxygen consumption in MIA PaCa‐2 A6 or NTC cells (*left*) and CFPAC‐1 CD147 or VC cells (*righ*t). (B) ATP production in MIA PaCa‐2/PANC‐1 A6 and NTC cells, as well as in CFPAC‐1 CD147 and VC cells. (C and D) ALDH^+^ subpopulation (C) and tumorsphere number (D) in CFPAC‐1 CD147 or VC cells treated with or without oligomycin A. (E) Activity of complex I in MIA PaCa‐2/PANC‐1 A6 or NTC cells, with rotenone serving as a control. (F–H) ALDH^+^ subpopulation (F), tumor sphere numbers (G), and mRNA levels of *NANOG* and *SOX2* (H) in CFPAC‐1 CD147 or VC cells with or without rotenone treatment. (I) Protein levels of *NDUFS1* and *CD147* in MIA PaCa‐2 A6 or NTC cells and CFPAC‐1 CD147 or VC cells. (J and K) ALDH^+^ subpopulation (J) and tumoursphere numbers (K) in CFPAC‐1 CD147 or VC cells with or without *NDUFS1* knockdown. CSC, cancer stem cell; NTC, non‐target control; VC, control vector.

Subsequently, we investigated whether the promotion of CSC stemness by *CD147* was due to the regulation of OXPHOS and ETC complex I. We observed that OCR was significantly lower in *CD147* knockdown A6 cells than in NTC, whereas it was markedly higher in *CD147* knock‐in cells than in VC (Figure 4A). These observations were further confirmed by measuring ATP levels and oxygen consumption (Figure 4B, Figure S6A). Moreover, the increase in the ALDH^+^ ratio and enhancement of tumor sphere numbers resulting from *CD147* overexpression were largely blocked by oligomycin A (Figure 4C, D). In addition, we found that *CD147* knockdown significantly decreased the activity of complexes I and II, but not that of complexes III and IV (Figure 4E, Figure S6B–D). Consequently, rotenone, an inhibitor of complex I, notably attenuated the effects of *CD147* overexpression on the ALDH^+^ CSC ratio, spherogenic capacity, and expression of stemness genes (Figure 4F–H). These results suggested that *CD147* promotes both ETC complex I and subsequent OXPHOS, which in turn mediates pancreatic CSC stemness.

### 2.4 CD147 Promotes NDUFS1‐Mediated Pancreatic CSC Stemness by Enhancing pSTAT3 Transcriptional Activity

We explored whether CD147 promotes the stemness of pancreatic CSCs by upregulating the complex I subunit, *NDUFS1*. We found that NDUFS1 protein levels changed as the levels of CD147 increased (Figure 4I). Functional analysis showed that *NDUFS1* knockdown significantly abolished the CD147‐promoted increase in the ALDH^+^ ratio and tumorsphere‐forming ability (Figure 4J–K). These results imply that CD147 promotes the NDUFS1‐mediated maintenance of pancreatic CSC stemness.

**FIGURE 5:**
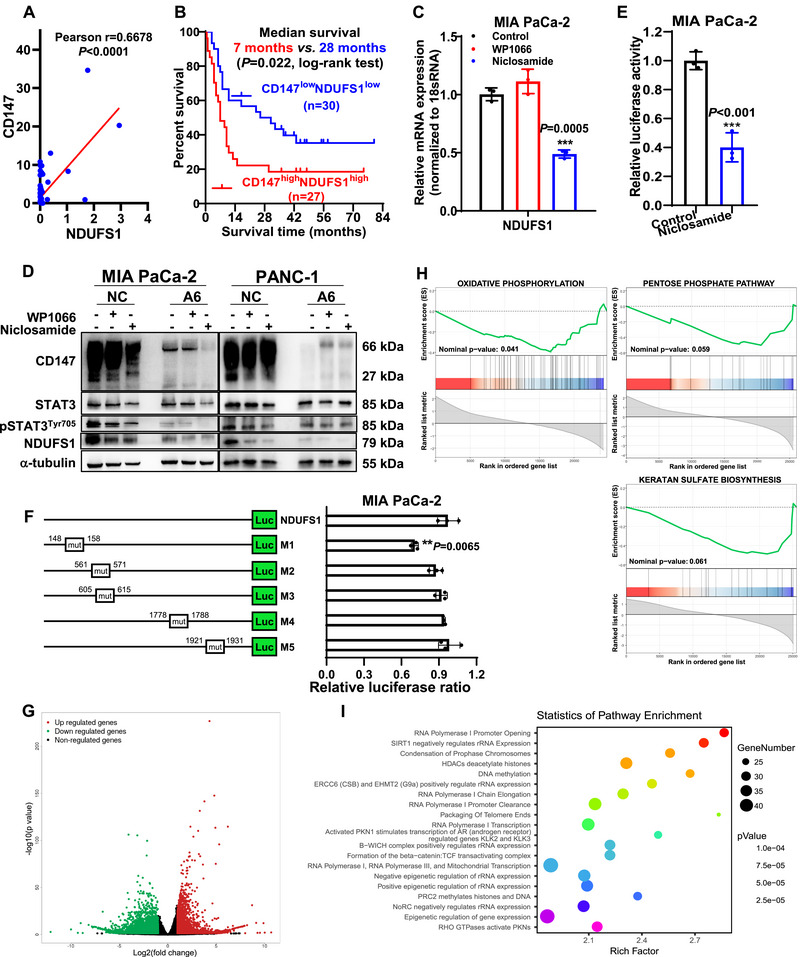
CD147 promotes NDUFS1 transcription via pSTAT3 and enhances NDUFS1‐mediated pancreatic CSC stemness through a metabolic epigenetic mechanism. (A) Correlation between CD147 and NDUFS1 expression. (B) Overall survival of 57 patients with low or high CD147/NDUFS1 co‐expression. (C) ND*UFS1* mRNA levels in cells treated with or without 1 µM WP1066 or 0.5 µM niclosamide for 24 h. (D) Protein levels of CD147, pSTAT3^Tyr705^, STAT3, and NDUFS1 in NC/A6 cells with or without 1 µM WP1066 or 0.5 µM niclosamide for 24 h. (E) ND*UFS1* transcriptional activity in cells treated with or without 0.5 µM niclosamide for 24 h. (F) Different mutated constructs of the endogenous *NDUFS1* promoter (M1–M5, *left*) and *NDUFS1* transcriptional activity in cells transfected with the indicated constructs (*right*). (G–I) Volcano plot (G), metabolic pathway GSEA (H), and pathway enrichment (I) of DEGs from transcriptome sequencing of A6 SP and NTC SP. CSC, cancer stem cell; GSEA, gene set enrichment analysis.

To conduct a more in‐depth exploration of the clinicopathological relationship between these two molecules, we performed a multiplex immunofluorescence assay on pancreatic cancer TMA samples from 99 patients. These results indicate a strong correlation between CD147 and NDUFS1 expression (Figure 5A, Figure S6E). Moreover, patients with a higher level of CD147 and NDUFS1 co‐expression exhibited significantly worse clinical outcomes, such as a higher tumor grade, more advanced AJCC and TNM stages, and shorter overall survival (Figure 5B, Table 2).

**TABLE 2:** Association of the CD147/ ndufs1 co‐expression with patient's clinicopathological features.

Clinicopathological features	CD147^low,^ ^a^ndufs1^low,^ ^a^	CD147^high,^ ^a^ndufs1^high,^ ^a^	*p* value^b^
Age (*n*, ≤ 60/> 60 years old)	18/12	12/15	**0.006**
Gender (*n*, male/female)	16/15	19/8	**0.000**
Tumor volume (*n*, > 35 cm^3^/< 35 cm^3^)	15/16	9/18	**0.016**
Tumor type (*n*, PDAC/ASCP and others)	22/9	23/4	**0.000**
Tumor grade (*n*, I‐II/II/II‐III/III/III‐IV/IV)	3/23/4/1/0/0	1/9/10/6/0/1	**0.000**
AJCC clinical grade (*n*, 1/2/3/4)	9/20/0/0	11/15/0/1	**0.010**
T stage (*n*, T1/T2/T3)	0/27/4	0/19/8	**0.012**
N stage (*n*, N0/N1)	11/18	17/8	**0.013**
M stage (*n*, M0/M1)	31/0	26/1	**0.001**
Metastasis (*n*, yes/no)	0/31	1/26	**0.001**
Lymph node invasion (*n*, yes/no)	11/18	16/8	0.077
Ki‐67 (*n*, < 5/5–10/10–20/20–30/> 30%)	3/7/6/7/6	4/7/6/5/4	0.686
P53 (*n*, < 5/5–20/20–40/40–60/60–80/> 80%)	12/4/4/4/2/3	10/1/3/1/6/5	0.183
OS (*n*, live/dead)	11/20	5/22	**0.000**

^a^ Low (negative to weak expression), high (moderate to strong expression).

^b^ Estimated by *χ* ^2^ test.

Our previous studies have shown that in pancreatic cancer cells, CD147 activates STAT3 signaling through its interacting protein, CD44s, thereby inducing cell proliferation and invasion [27]. STAT3 also plays a vital role in maintaining pancreatic CSC stemness [28]. Based on these findings, we investigated whether CD147 regulates NDUFS1 via the STAT3 signaling. Our results showed that niclosamide, a specific inhibitor of STAT3 transcription, but not WP1066, effectively decreased NDUFS1 mRNA and protein levels. When combined with CD147 knockdown, NDUFS1 protein levels were further reduced (Figure 5C, D). The dual‐luciferase reporter assay showed that niclosamide significantly decreased *NDUFS1* transcription by 60% (Figure 5E). Using JASPAR (https://jaspar.genereg.net/) to predict the binding site of STAT3 to the *NDUFS1* promoter, we constructed five truncated versions of the *NDUFS1* promoter. Luciferase activity was significantly reduced in the M1 mutant, suggesting that the promoter region between 148 and 158 bp was indispensable for the STAT3‐mediated regulation of *NDUFS1* transcription (Figure 5F). Taken together, our results indicated that CD147 promotes STAT3‐mediated *NDUFS1* transcription, which consequently enhances complex I activity and pancreatic CSC stemness.

### 2.5 CD147‐NDUFS1 Signaling Mediates Pancreatic CSC Stemness via SIRT1/DNMT1‐Activated PAX2 Hypomethylation

To investigate how *CD147‐NDUFS1* promotes stemness, we performed transcriptome sequencing of pancreatic CSCs derived from SP cells enriched with *CD147* knockdown. The KEGG pathway analysis of the DEGs revealed that the two most enriched pathways (ranked by gene count) were signal transduction and energy metabolism (Figure 5G, Figure S6F). Notably, among the top six metabolic pathways, oxidative phosphorylation was the only pathway that was significantly downregulated following *CD147* knockdown (Figure 5H, Figure S6G). Reactome analysis identified key epigenetic regulatory pathways, such as SIRT1 negatively regulating rRNA expression, HDACs deacetylating histones, and DNA methylation, among the top five most enriched pathways (Figure 5I). Based on these findings, we explored the meta‐oboepigenetic mechanism by which *CD147‐NDUFS1* maintains pancreatic CSC stemness.

**FIGURE 6:**
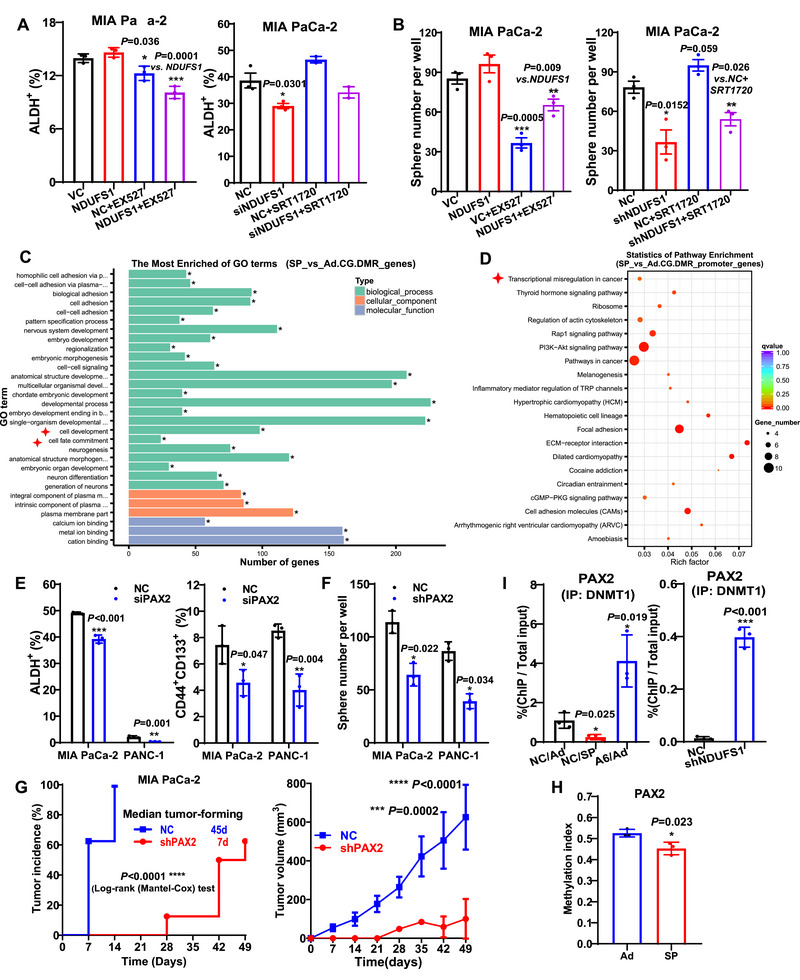
CD147‐NDUFS1 signaling promotes pancreatic CSC stemness via SIRT1/DNMT1‐mediated *PAX2* hypomethylation. (A and B) ALDH^+^ subpopulation (A) and tumorsphere numbers (B) in NDUFS1 knockin (NDUFS1) or VC cells treated with or without 1 µM EX527, and in *NDUFS1* knockdown (shNDUFS1) or NC cells treated with or without 1 µM SRT1720. (C and D) GO (C) and pathway (D) enrichment analyses of differentially methylated genes based on bisulfite sequencing of SP and Ad cells. (E) ALDH^+^ and CD44^+^CD133^+^ subpopulations in MIA PaCa‐2/PANC‐1 cells without (NC) or with *PAX2* interference (siPAX2). (F) Tumorsphere numbers in MIA PaCa‐2/PANC‐1 cells without (NC) or with *PAX2* knockdown (shPAX2). (G) Tumor incidence (*left*) and volume (*right*) in nude mice subcutaneously implanted with 2 × 10^6^ MIA PaCa‐2 cells without (NC) or with *PAX2* knockdown (shPAX2). (H) Methylation index of the *PAX2* promoter in SP and Ad cells. (I) ChIP assay of the binding of DNMT1 to the *PAX2* promoter in SP, A6, and NC/Ad cells (*left*), and in shNDUFS1 and NC cells (*right*). CSC, cancer stem cell; GO, gene ontology.

Complex I (NADH: ubiquinone oxidoreductase) is crucial for NAD^+^ regeneration, prompting us to investigate SIRT1, an NAD^+^‐dependent deacetylase that regulates stemness maintenance and cellular metabolism, and is significantly downregulated in transcriptome data. Notably, pancreatic CSCs exhibited higher NAD^+^ and SIRT1 expression levels than non‐CSCs (Figure S7A–C). Strikingly, pharmacological modulation of SIRT1 activity revealed that the SIRT1 inhibitor (EX527) reduced, whereas its activator (SRT1720) increased, NDUFS1‐driven enhancements in the ALDH^+^ CSC ratio, spherogenesis capacity, and stemness gene expression (Figure 6A, B, Figure S7D). Furthermore, *CD147* knockdown reduced NAD^+^ levels and SIRT1 expression, whereas *SIRT1* silencing significantly reduced the CD147‐promoted spherogenesis (Figure S7E–G). Together, these results established that SIRT1 is a key downstream effector of CD147/NDUFS1‐mediated stemness maintenance in pancreatic CSCs.

Transcriptome analysis revealed significant enrichment of the DNA methylation pathway among the DEGs in *CD147* knockdown CSCs. To further explore this finding, we performed bisulfite sequencing to compare the DNA methylation profiles between CSCs and non‐CSCs. Functional annotation of differentially methylated genes demonstrated their predominant enrichment in cell development and cell fate commitment, whereas pathway analysis highlighted significant enrichment in transcriptional misregulation in cancer (Figure 6C, D). By intersecting genes related to cell‐fate commitment with established human stem cell transcription factors (Table S4), we identified significant methylation alterations in the promoter regions of *PAX2* and *HOXB2*. However, in pancreatic CSCs, only *PAX2* was substantially upregulated at both mRNA and protein levels (Figure S7H, I). Notably, *PAX2* silencing significantly reduced the CSC population, suppressed tumorsphere formation, and blunted both tumor initiation and tumor growth in nude mice, collectively establishing its essential role in sustaining pancreatic CSC stemness (Figure 6E–G, Figure S7J). Furthermore, methylation index analysis revealed significantly reduced methylation levels at the *PAX2* promoter in CSCs relative to non‐CSCs (Figure 6H), suggesting that PAX2 upregulation via promoter hypomethylation appeared to be a key mechanism underlying the CD147‐NDUFS1 SIRT1‐mediated maintenance of pancreatic CSC stemness.

We subsequently investigated how the CD147‐NDUFS1‐SIRT1 axis regulates PAX2 expression and CSC stemness. *CD147* overexpression significantly upregulated *PAX2* expression, whereas *SIRT1* interference or STAT3 pharmacological inhibition effectively downregulated *PAX2* expression (Figure S7K–L). Moreover, when STAT3 inhibition was combined with CD147 knockdown, PAX2 protein levels were further reduced (Figure S7M). Notably, both siRNA‐mediated SIRT1 silencing and treatment with SIRT1 modulators (EX527/SRT1720) effectively reversed the upregulation of *PAX2* induced by CD147 or NDUFS1 overexpression (Figure S7K, N). Furthermore, NAD^+^ depletion with FK866 completely reversed the effects of NDUFS1 overexpression, including enhanced SIRT1 activity, elevated PAX2 expression, and increased stemness potentials. Conversely, exogenous NAD^+^ supplementation restored both SIRT1 activity and PAX2 expression, rescuing the impaired stemness caused by NDUFS1 knockdown (Figure S8A–E). Clinicopathological analysis of pancreatic cancer specimens showed a marked elevation in *CD147*, *NDUFS1, SIRT1*, and *PAX2* mRNA levels in tumor tissues compared with those in adjacent normal tissues, further supporting the pathophysiological relevance of this regulatory axis in human pancreatic cancer (Figure S8F).

To identify the specific enzymes responsible for *PAX2* methylation, we systematically evaluated three possible DNA methyltransferases (*DNMT1*, *DNMT3A*, and *DNMT3B)* using RNA. The results showed that *DNMT1* knockdown elicited the most significant upregulation of both PAX2 mRNA and protein levels, suggesting its predominant role in PAX2 repression (Figure S8G, H). We further examined whether *PAX2* expression was regulated by DNMT1 using a ChIP assay. Our results showed that DNMT1 binding to the *PAX2* promoter was significantly lower in SP CSCs than in Ad non‐CSCs; however, DNMT1 binding was significantly higher in *CD147* knockdown A6 non‐CSCs than in NTC non‐CSCs and was also higher in *NDUFS1* knockdown cells than in NC cells (Figure 6I). Notably, pharmacological modulation of SIRT1 activity using EX527 or SRT1720 dynamically regulated the DNMT1 acetylation status (Figure S8I). Collectively, our results indicate that CD147 enhances STAT3 signaling and *NDUFS1* transcription, which in turn increases complex I activity in OXPHOS, and the increased NAD^+^ produced during OXPHOS activates SIRT1 and decreases DNMT1 enzymatic activity, leading to hypomethylation of the *PAX2* promoter and enhanced pancreatic CSC stemness.

## 3 Discussion

This study advances our understanding of the metabolic‐epigenetic regulation of CSC stemness by three critical findings: (i) revealing a previously undescribed role of mitochondrial complex I activity in maintaining pancreatic CSC stemness; (ii) identifying a novel NDUFS1‐derived metabolic epigenetic signaling (SIRT1‐DNMT1‐PAX2) that couples OXPHOS to stemness transcription; and (iii) demonstrating that CD147 transcriptionally activates *NDUFS1* to enhance complex I activity, highlighting its potential as a therapeutic target to disrupt OXPHOS‐dependent stemness. Overall, our study describes a new mechanism for maintaining CSC stemness, in which CSCs exhibit enhanced OXPHOS by upregulating mitochondrial complex I activity via the CD147‐NDUFS1 axis, thereby sustaining stemness through an integrated metabolic‐epigenetic circuitry centered on SIRT1/DNMT1‐mediated PAX2 activation.

Studies have examined the metabolic profiles of pancreatic CSCs, highlighting their remarkable adaptability in utilizing different substrates under varying conditions. For example, glutamine can serve as a fuel for CD9^high^ pancreatic tumor‐initiating cells (TICs), effectively driving tumor growth [29]. Furthermore, fatty acid synthesis and the mevalonate pathway have emerged as alternative metabolic routes essential for the survival of pancreatic CSCs [30]. However, in this study, our data suggested that glucose‐derived OXPHOS fueled pancreatic CSCs. This conclusion is supported by two pieces of evidence: pancreatic CSCs exhibit a significantly higher rate of glucose uptake, and UK 5099, an OXPHOS‐specific inhibitor, effectively suppresses this glucose‐dependent effect. Regardless of whether the energy source is glucose, glutamine, or fatty acids, the main contributor to pancreatic CSC stemness may be the terminal stage of biological oxidation, OXPHOS.

As the first and largest component of the OXPHOS system, mitochondrial complex I transfers two electrons from NADH to quinone and transports four protons across the membrane, providing 40% of the total proton motive force [31, 32]. Previous studies have reported that complex I inhibitors (such as metformin, phenformin, rotenone, NV‐128, etc.) are effective as selective anti‐CSC agents for the treatment of pancreatic, breast, and ovarian cancers [9, 33, 34, 35]. Defects in complex I lead to ROS accumulation and decreased NAD^+^ levels [32]. However, the precise role of complex I in regulating CSC stemness remains poorly understood. A major finding of our study was that the markedly elevated complex I activity in pancreatic CSCs sustained the stemness of pancreatic CSCs. Elucidating the role of complex I in regulating stemness greatly broadens the potential application of existing drugs. More importantly, beyond the well‐known nuclear‐controlled ATP production, we provided evidence that mitochondria function as key signaling organelles by controlling stem cell fate through metaboloepigenetic retrograde signaling in the nucleus [36]. Therefore, targeting a key player or regulator in this mito‐nuclear communication system holds promise as an effective strategy for eradicating pancreatic CSCs.

NDUFS1 appears to be a key player in mitonuclear communication for controlling pancreatic CSC stemness. Although its role in mitochondrial dysfunction‐related disorders (such as cardiac hypertrophy and diabetic cardiomyopathy) has been well documented [37, 38], its function in cancer exhibits a context‐dependent duality, acting as either a tumor suppressor or an oncogene. High *NDUFS1* expression is associated with a favorable prognosis in ovarian cancer, clear cell renal cell carcinoma, and non‐small cell lung cancer [39, 40, 41, 42]. In lung adenocarcinoma and gastric cancer, NDUFS1 suppresses tumor progression by inhibiting invasion, migration, and epithelial‐mesenchymal transmission [43, 44]. Conversely, in breast cancer [45], hepatocellular carcinoma [46], and colorectal cancer [47], elevated NDUFS1 expression promotes cell proliferation and tumorigenesis. Notably, in glioblastoma stem cells (GSCs), NDUFS1 interacts with the oncostatin M receptor (OSMR) to regulate respiration and confer radiation resistance [48]. Despite these advances, the role of NDUFS1 in the maintenance of CSC stemness remains unclear.

In this study, we found that NDUFS1 is highly expressed in pancreatic CSCs and is essential for sustaining their stemness and tumorigenicity, indicating that mitochondrial metabolic genes can affect the functional status of pancreatic CSCs. In particular, we found that NDUFS1‐composed mitochondrial complex I in OXPHOS enhanced pancreatic CSC stemness by increasing NAD^+^ production, which in turn activated SIRT1‐mediated acetylation of DNMT1 (previously proposed as a potential target for pancreatic CSCs [49]), ultimately upregulating the expression of the stemness‐related gene PAX2 via DNMT1‐directed hypomethylation. Unlike the evolutionarily conserved core pluripotency transcription factors Nanog, SOX2, and OCT4, which are essential for maintaining the pluripotent state and self‐renewal capacity of diverse stem cells, PAX2 has traditionally been recognized as a lineage‐specific transcription factor. It guides progenitor cells toward kidney, optic, or otic fates after the cells exit the pluripotent state. Our study has now uncovered a non‐canonical, pancreatic cancer‐restricted role of PAX2 in governing stemness. In this context, PAX2 is activated and switched on by a specific metabolic‐epigenetic signaling pathway. Once activated, PAX2 is sufficient to maintain the stemness potential and self‐renewal capacity of pancreatic CSCs without directly altering the core pluripotency network.

Bioinformatic analysis based on the stemness index indicated that *CD147*, an upstream regulator of *NDUFS1*, was significantly upregulated in pancreatic CSCs and promoted complex I‐induced stemness potential by enhancing STAT3‐mediated *NDUFS1* transcription. Although the NDUFS1 expression alone did not serve as a prognostic factor, its prognostic significance became prominent when combined with CD147 expression, suggesting that the clinical relevance of NDUFS1 is unveiled only in the context of its upstream regulator, CD147, highlighting the importance of evaluating both markers. The NDUFS1^high^CD147^high^ signature may define a unique molecular subtype of pancreatic cancer characterized by enhanced stemness and increased aggressiveness, resulting in significantly worse clinical outcomes. These survival data mirror our functional results, demonstrating that CD147 endows pancreatic cancer cells with stem cell‐like properties through NDUFS1, offering compelling clinical evidence for the co‐targeting of this axis. Collectively, we identified a new function of NDUFS1 in sustaining pancreatic CSC stemness, along with its upstream regulators, transcription factors, and downstream metaboloepigenetic signaling, which determine stemness potential.

We have previously reported that CD147 promotes the proliferation and invasion of pancreatic cancer cells [27, 50]. Moreover, the anti‐CD147 antibody was found to suppress the stemness of pancreatic CSCs [26]. However, no previous studies have explored the direct effects of CD147 on pancreatic CSCs. In our study, we provide direct evidence that CD147 enhances the self‐renewal ability and tumorigenicity of pancreatic CSCs. This finding supports the previous opinion that CD147 is a potential therapeutic target. Furthermore, we identified a novel role for CD147 in regulating the ETC of OXPHOS via the regulation of NDUFS1 transcription. In contrast to previous studies that focused on CD147's role in promoting glycolysis and fatty acid metabolism, we propose that the metabolic function of CD147 is context dependent [51, 52, 53, 54]. From the perspective of translational medicine, an anti‐CD147 agent (metuximab) developed in our laboratory has been shown to prevent tumor recurrence after liver transplantation or radiofrequency ablation [55, 56]. It has also been demonstrated to enhance the chemosensitivity of pancreatic CSCs and NSCLC [26, 57]. Further investigation of the role of CD147 in pancreatic CSCs could lay the foundation for broader clinical applications of metuximab.

This study reveals a novel mechanism by which pancreatic CSC stemness maintenance driven by complex I and downstream metabolo‐epigenetic circuitry. However, several limitations warrant consideration. First, the reliance on cell lines and xenografts necessitates validation in more physiological models such as patient‐derived organoids or genetically engineered mice. Second, although CD147 and NDUFS1 co‐expression correlates with poor prognosis, targeting mitochondrial complex I risks off‐target effects due to its essential role in normal cell energetics. Finally, while OXPHOS is central to stemness regulation in our model, the contribution of alternative energy sources such as glutamine or fatty acids to stemness under varying nutrient conditions remains unclear. Addressing these points in future will improve understanding of metabolic‐epigenetic crosstalk in CSCs and advance therapeutic strategies against pancreatic cancer stemness.

## 4 Conclusions

In this study, we discovered that the upregulated activity of mitochondrial complex I in pancreatic CSCs sustains their stemness potential through NDUFS1‐mediated metaboloepigenetic signaling, which is initiated by the high expression of CD147 and its promotion of NDUFS1 transcription. Our study provides evidence that mitochondria function as key signaling organelles that control stem cell fate through retrograde signaling to the nucleus, in that NDUFS1 mediates SIRT1‐DNMT1 metaboloepigenetic signaling. Blocking a key regulator in the mitonuclear communication, for example, targeting CD147, may be a novel therapy for pancreatic cancer.

## 5 Materials and Methods

### 5.1 Patient‐Derived Xenograft (PDX) Model and Tumor Cell Separation

Patient‐derived pancreatic tumor xenograft model PDX 374 was obtained from Xi'an Lide Biotechnology. Briefly, tumor tissues were cut into small pieces (4 × 2 × 2 mm^3^) and subcutaneously implanted into the backs of 5‐ to 6‐week‐old female athymic BALB/c nude mice. The mice were euthanized when the maximum tumor diameter was < 1 cm. The subcutaneous tumors were then excised using blunt dissection with forceps and scissors and weighed. Each gram of tumor tissue was placed in 10 mL of cell dissociation buffer (Dulbecco's modified Eagle's medium [DMEM] supplemented with 10% fetal bovine serum [FBS], 1% mycillin, 200 U/mL collagenase type IV, and 0.6 U/mL dispase), and then minced. The cell suspension was transferred into a 50‐mL conical tube, vortexed at maximum speed for 1 min, and incubated at 37°C for 2 h with additional 1‐min vortexing every 20 min. The samples were then passed through a 70‐µm filter for subsequent experiments.

### 5.2 Tumorsphere Culture and Passaging

Cells were suspended in DMEM/F12 serum‐free medium containing 1% N2, 2% B27, 15% glucose, 10 ng/mL human bFGF, 10 ng/mL EGF, 5 µg/mL insulin, 5 µg/mL β‐mercaptoethanol, and 0.2% heparin, and seeded into 6‐well ultra‐low attachment plates (Corning, NY) in triplicate at a density of 6 × 10^3^ or 2 × 10^4^ cells per well. The cells were then cultured for 10–14 days to allow the formation of tumorspheres, which were subsequently quantified under an inverted microscope (Olympus).

For tumorsphere passaging, spheroids were collected with a 40‐µm Cell Strainer (Corning, San Jose, CA), separated into a single cell suspension using TrypLE (Thermo Fisher Scientific, Waltham, MA), and re‐seeded for culture over an additional 10–14 days. Tumorspheres passaged three times were used to obtain stem‐like cells (SP), and adherent cells (Ads) were considered non‐stem cells. Tumorsphere formation efficiency (SFE) was calculated as the percentage of tumorspheres with diameters >70 µm relative to the original number of cells.

For tumorsphere formation from single cells, one cell per well was seeded into 96‐well ultra‐low attachment plates (Corning), and the tumorsphere medium was renewed every 7 days for 1 month.

### 5.3 ALDEFLUOR Assay and Sorting of ALDH+/− Subpopulations

ALDH^+^ populations were identified and sorted using the ALDEFLUOR kit (Stem Cell Technologies) according to the manufacturer's instructions and a previously described method [26]. Briefly, 1 × 10^6^ cells were incubated for 45 min at 37°C in ALDEFLUOR assay buffer containing 1 µM BAAA. To establish the sorting gate, a control sample was treated with 50 mM of the ALDH inhibitor diethylamino benzaldehyde (DEAB). After incubation, cells were resuspended in a solution of 2% FBS in HBSS containing 1 µg/mL 7‐AAD for live‐cell discrimination. Using the DEAB‐treated control to define the negative population, ALDH^+^ and ALDH^—^ cells were isolated with a FACS Aria III cell sorter (BD Biosciences), achieving a sort purity of > 90%.

### 5.4 Oxygen Consumption Rate (OCR) and Extracellular Acidification Rate Measurement (ECAR)

Cellular OCR and ECAR were measured using an XF96 Flux Analyzer (Agilent Technologies). Briefly, cells were seeded into an XF96 cell culture microplate at 1 × 10^4^ cells per well and cultured overnight at 37°C in a CO_2_ incubator. When the cells reached 80%‒90% confluence, the medium was replaced with the XF assay medium, and the cells were incubated at 37°C in a 0% CO_2_ incubator for 45 min. The effects of different drugs were then determined. A mitochondrial stress test was performed using the XF base medium supplemented with 1 M glucose, 100 mM pyruvate, and 200 mM glutamine. The test drugs and their concentrations were 0.75 µM oligomycin, 0.5 µM FCCP, and 2 µM antimycin A. A glycolysis stress test was performed using the XF base medium supplemented with 2 mM glutamine. The test drugs and their concentrations were 10 mM glucose, 1 µM oligomycin, and 50 mM 2‐deoxy‐D‐glucose (2‐DG).

OCR was determined using a Cayman Oxygen Consumption Rate Assay Kit. Briefly, 1 × 10^4^ cells/well were seeded into a 96‐well plate and incubated overnight. Then, 150 µL of fresh medium and 10 µL of the MitoXpress‐Xtra solution were added to replace the spent medium. Subsequently, each well was covered with 100 µL of HS Mineral Oil. The absorbance was immediately read at 650 nm for 2 h using a spectrophotometer (BioTek, Winooski, VT, USA). The OCR was calculated as (mOD1 − mOD2)/*T*, where mOD1 is the initial absorbance, mOD2 is the final absorbance, and *T* is time in minutes.

### 5.4 CD44^+^CD133^+^ Subpopulation Analysis and Sorting of CD44^+/−^ Subpopulations

For CD44^+^CD133^+^ subpopulation analysis, 1 × 10^6^ cells were incubated with 1 µL each of mouse anti‐CD133‐PE and mouse anti‐human CD44‐APC antibodies in PBS at 4°C for 30 min. An isotype‐matched mouse immunoglobulin was used as a negative control. Samples were analyzed using a FACS Calibur Flow cytometer, and data were analyzed using the CellQuest software (BD Biosciences, San Jose, CA, USA). For cell sorting, cells were stained with the indicated antibodies and suspended in 2% FBS/HBSS with 1 µg/mL 7‐AAD for viable cell gating. Positive and negative cells were sorted via flow cytometry (BD FACSAria III). The sorted cells had a purity of > 90%.

### 5.5 Mitochondrial Complex I/II/Ш/IV Activity

The activities of mitochondrial complexes in the ETC were measured using an Enzyme Activity Microplate Assay kit (Abcam, Cambridge, MA). Briefly, 1 × 10^7^ cells were lysed, and total protein was quantified using a BCA Protein Assay kit (Beyotime Biotechnology, Shangahai, CN). The lysed cells were then incubated at room temperature for 2–3 h in a 96‐well microplate coated with monoclonal antibodies specific to complex I, II, Ш, or IV. Subsequently, specific substrates were added. Absorbance at 450, 600, 450, or 550 nm was measured continuously using a spectrophotometer (Biotek, Winooski, VT) for 30 min (complex I), 2 h (complex II), 15 min (complex Ш), or 2 h (complex IV). The reaction rate of each complex (mOD/min) was calculated as (mOD_1_ − mOD_2_)/*T*, where mOD_1_ is the initial absorbance, mOD_2_ is the final absorbance, and *T* is time in minutes.

## Author Contributions

L.L., Z.‐N.C., and H.‐Y.C. conceived the study and participated in study design, performance, coordination, and manuscript writing. X.‐Y.F., W.L., Y.S., B.‐Q.X., H.W., R.‐F.T., Z.‐C.D., J.F., J.‐R.L., X.‐X.S., B.W., L.‐J.W., K.W., S.‐J.W., and X.‐M.Y. obtained the samples, performed the experiments, interpreted the data, and prepared the figures. All authors have reviewed and approved the final manuscript.

## Funding

This study was supported in part by grants from the National Natural Science Foundation of China (#31571469 and #82173244), Innovation Capacity Support Program of Shaanxi Province—Science and Technology Resources Open Sharing Platform (2025JC‐GXPT‐042), State Key Laboratory of Holistic Integrative Management of Gastrointestinal Cancers (CBSKL2022ZZ09 and 2025GTKP007), General Project of Natural Science Basic Research Program of Shaanxi (#2022JQ‐874), and The Key Research and Development Program of Shaanxi (2023‐YBSF‐176).

## Ethics Statement

All animal experimental protocols in this study were approved by the Animal Care and Use Committee of Fourth Military Medical University (Approval No. 2022‐NTSCMM‐ID003).

## Conflicts of Interest

The authors declare no conflicts of interest.

## Supporting information




**Supporting File 1**: Mco270678‐sup‐0001‐SuppMat.Pdf

## Data Availability

The datasets used and/or analyzed in the current study are available from the corresponding author upon reasonable request.
