# Supplementary material for: NDUFS1‐Mediated Mitochondrial Complex I Activity Maintains Pancreatic Cancer Stemness by Promoting PAX2 Hypomethylation
Source: MedComm (2020). 2026 Mar 18;7(4):e70678. doi: 10.1002/mco2.70678 (PMC13042754; doi:10.1002/mco2.70678)
Supplement: Supplementary file 1 — Supporting File 1: Mco270678‐sup‐0001‐SuppMat.Pdf [file MCO2-7-e70678-s001.pdf]

NDUFS1-mediated mitochondrial complex I activity maintains pancreatic cancer stemness by promoting PAX2 hypomethylation

Running title: Complex I activity maintains pancreatic cancer stemness

Xin-Yu Fan[1, #], Wen Li[1, #], Ying Shi[1, #], Bao-Qing Xu[1,2, #], Hao Wang[1], Ruo-Fei Tian[1], Zi-Chuan Duan[1], Jing Fan[1], Jia-Rong Liu[1], Xiu-Xuan Sun[1], Bin Wang[1], Li-juan Wang[1], Ke-Wang[1], Shi-jie Wang[1], Xiang-Min Yang[1], Hong-Yong Cui[1, *], Zhi-Nan Chen[1, *], Ling Li[1, *]

[1] Department of Cell Biology, National Translational Science Center for Molecular Medicine, State Key Laboratory of Holistic Integrative Management of Gastrointestinal Cancer, State Key Laboratory of New Targets Discovery and Drug Development for Major Diseases, Fourth Military Medical University, Xi'an, China;

[2] The 900th Hospital of Joint Logistics Support Force, Fuzhou, 350025, China.

Corresponding author:

Zhi-Nan Chen, Department of Cell Biology, National Translational Science Center for Molecular Medicine, State Key Laboratory of Holistic Integrative Management of Gastrointestinal Cancers, State Key Laboratory of New Targets Discovery and Drug

Development for Major Diseases, Fourth Military Medical University, Xi'an 710032, China. E-mail: znchen@fmmu.edu.cn

Ling Li, Department of Cell Biology, National Translational Science Center for Molecular Medicine, State Key Laboratory of Holistic Integrative Management of Gastrointestinal Cancers, State Key Laboratory of New Targets Discovery and Drug Development for Major Diseases, Fourth Military Medical University, Xi'an 710032, China. E-mail: liling25@fmmu.edu.cn

Hong-Yong Cui, Department of Cell Biology, National Translational Science Center for Molecular Medicine, State Key Laboratory of Holistic Integrative Management of Gastrointestinal Cancer, State Key Laboratory of New Targets Discovery and Drug Development for Major Diseases, Fourth Military Medical University, Xi'an 710032, China. E-mail: cuihongyong@fmmu.edu.cn

Supplementary Materials and Methods

Cells and constructs

Three human pancreatic cancer cell lines (MIA PaCa-2, PANC-1, and CFPAC-1) and human embryonic kidney cells (HEK293) were purchased from American Type Culture Collection and cultured in Dulbecco's modified Eagle medium (DMEM) with 10% fetal bovine serum (HyClone). All cell lines tested negative for mycoplasma and their identities were confirmed by short tandem repeat (STR) profiling.

An *NDUFS1* overexpression construct was established by subcloning human *NDUFS1* cDNA into a pcDNA3.1 expression vector. NDUFS1 lentiviral shRNA, NDUFS1-siRNA, SIRT1-siRNA, DNMT1-siRNA, DNMT3A-siRNA, DNMT3B-siRNA, and PAX2-siRNA were obtained from Genepharma (Shanghai, China; **Table S5**). Silencer siRNAs were used as negative controls (NCs). *CD147* pLKO.1, pGIPZ lentiviral shRNA (A6), and pGIPZ empty vector were obtained from Open Biosystems. The MISSION® Non-Target shRNA Control Vector (pLKO.1-NTC) was obtained from Sigma-Aldrich. A *CD147* overexpression construct was established by subcloning the human *CD147* cDNA into a GV341 lentiviral expression vector (Genechem, Shanghai, China).

Establishment of stable cell sublines

NDUFS1 or CD147 lentiviral shRNAs and their negative control (NTC or NC) shRNAs, NDUFS1 lentiviral cDNA or CD147 cDNA and their vector controls (VCs) were transfected into pancreatic cancer cells. Stable cell subclones were selected by

adding culture medium containing 4 to 6 μg/mL of puromycin, and expression was determined using qPCR and immunoblotting.

Tissue microarray

A tissue microarray (TMA) constructed from paraffin-embedded tissue blocks was purchased from Shanghai Outdo Biotech Company. The chip was built from tissues of 99 pancreatic cancer patients who were hospitalized between September 2004 and December 2008. All patients underwent primary surgical interventions. Clinicopathological data were collected from the patients' medical records, and included age at diagnosis, tumor volume, type and grade, TNM stage, AJCC stage, overall survival (**Table S6**). The study protocol was approved by the ethics committee of The Fourth Military Medical University (KY20213199-1).

Bioinformatics analysis

Three gene expression microarray datasets (GSE16515, GSE32676, GSE102238) from the Gene Expression Omnibus (GEO) database and an RNA-seq dataset (TCGA-PAAD) from the Cancer Genome Atlas database (TCGA) database were analyzed to identify stemness-related differentially expressed genes (DEGs) in pancreatic cancer. The DEGs between cancer and normal tissues were identified using the *t-test* for GEO data and the edgeR package for TCGA data. Additionally, tumor samples from TCGA-PAAD were divided into high- and low- stemness groups based on the median level of stemness index. Stemness related genes were then analyzed using edgeR with a significance threshold of P value < 0.05 and $|\log2FC| > 1.2$.

Furthermore, the identified stemness related genes were intersected with the

DEGs obtained from both the GEO and TCGA datasets (GSE16515, GSE32676, GSE102238 and TCGA-PAAD) to determine the stemness-related DEGs. Finally, we subjected these stemness-related DEGs to Protein-Protein Interaction (PPI) Network analysis and Gene Ontology (GO) enrichment analysis via the STRING database (https://string-db.org).

The GEPIA platform was applied to analyzing the NDUFS1 expression profiling and patient overall survival (OS), in which database 179 pancreatic cancer samples and 171 normal pancreatic tissue samples were included. Dot maps of selected genes were generated. And, median levels of NDUFS1 expression were set as the threshold for high and low expression.

The Kaplan-Meier Plotter database was also used to identify the association between NDUFS1 expression and OS, in which analysis hazard ratios (HRs) with corresponding 95% confidence intervals (CIs) and log-rank P-values were calculated.

ATP measurements

Intracellular ATP was measured using an ATP Assay kit (Beyotime Biotechnology, Beijing, China). Briefly, cells were prepared in a lysis buffer, and total protein was quantified using a BCA Protein Assay kit (Beyotime Biotechnology). Then, a 20 μL sample from the lysis buffer was added into 100 μL of the ATP detection buffer, and chemiluminescence was measured using a luminometer (Promega, Madison, WI), and ATP levels was determined using a standard curve.

Glucose uptake and lactate production

Glucose uptake was analyzed by using a Glucose Assay kit (Robio, China). 5×10^5 cells per well were seeded into 6-well plates and cultivated for 24 h. Then 10 μL of culture supernatant was collected and added into 1 mL of an assay solution, incubated at 37°C for 15 min, and the absorbance was then measured at 505 nm.

Lactate production was measured using a Lactate Assay kit (Jiancheng, China). 5×10^5 cells per well were seeded into 6-well plates and cultivated for 24 h. Then 20 μL of culture supernatant was added into 1 mL of an assay solution, and incubated at 37°C for 10 min. Finally, 2 mL of a termination solution was added and the absorbance was measured at 530 nm.

Real-time quantitative PCR and qPCR array

Total cellular RNA was isolated using Trizol, reverse transcribed into cDNA, and then amplified by a Stratagene Mx3005P Multiplex Quantitative PCR system (Agilent Technologies, Santa Clara, CA) using appropriate primers (**Table S5**), as previously described[22]. The relative mRNA level was calculated as $2^{-(Ct[HKG] - Ct[GOI])}$, where Ct is the cycle threshold, HKG is the house-keeping gene (18s RNA), and GOI is the gene of interest.

Human Mitochondrial Energy PCR Array (PAHS-008Z) experiments were performed according to the protocol from Qiagen (Duesseldorf, Germany). Briefly, total RNA was isolated using Total RNA Kit II (Omega, USA) and then reverse-transcribed using the PrimeScript RT Reagent kit (TaKaRa, Japan). The cDNA was mixed with $2\times RT^2$ TB Green qPCR Master Mix and ddH_2O. The qPCR was then performed using a QuantStudio 7 Flex quantitative PCR system according to

the RT^2 Profiler PCR Array instructions using the following protocol: 95°C for 10 min and then 40 cycles of 95°C for 15 s and 60°C for 1 min. The data were normalized relative to HKGs (*ACTB*, *B2M*, *HPRT1*, *RPLP0*) by calculating the ΔCt for each gene of interest, as above. Fold changes of gene expression, scatterplots, and heatmaps were analyzed and generated using the RT^2 PCR array data analysis website (https://geneglobe.qiagen.com/us/analyze).

Immunoblotting

Whole cell lysis was performed using a phospho-RIPA buffer (1 M Tris-HCl at pH 7.5, 5 M NaCl, 0.01% NP-40, 0.5 M EGTA, and 10% SDS) that was supplemented with a complete EDTA-free protease inhibitor cocktail (Roche, Indianapolis, IN). After quantitation, protein samples were separated using sodium dodecyl sulfate-polyacrylamide gel electrophoresis (SDS-PAGE) gels and transferred onto polyvinylidene fluoride (PVDF) membranes. For immunoblotting, the membranes were first blocked in a 5% BSA/PBS solution at room temperature for 1 h. Then, they were incubated with the primary antibody and with horseradish peroxidase-conjugated anti-mouse or anti-rabbit IgG. Protein signals were detected using Enlight Western Blot ECL Reagents (Engreen, Beijing, China), with α-tubulin as a loading control.

Animal studies

Briefly, 5-6 week-old female athymic BALB/c nude mice or NOD-SCID mice were obtained from Silaikejingda Experimental Animal Co., Ltd (Hunan, China). Mice were given subcutaneous inoculations into both flanks with 1×10^6 PANC-1

shNDUFS1 or NC cells (n=10), 500,000 MIA PaCa-2 $ALDH^+$/$ALDH^-$ shNDUFS1 or NC cells (n=9), 5,000 to 500,000 MIA PaCa-2 $ALDH^+$ shNDUFS1 or NC cells (n=5) or 50 to 5000 PANC-1 shCD147 A6 or non-target control (NTC) cells (n=10). Tumor formation was monitored for 6 to 12 weeks. Tumors were measured using a caliper, and volume was calculated as (Length × $Width^2$)/2. All animal studies were approved by the Institutional Animal Care and Use Committee of The Air Force Medical University (Approval No. 2022-NTSCMM-ID003).

Multiplex immunofluorescence assay

The multiplex immunofluorescence assay was performed by Shanghai Outdo Biotech Company. First, the pancreatic cancer tissue microarray was dewaxed, and then antigen retrieval was performed using a citrate buffer. The tissues were then blocked in 5% goat serum, and sequentially incubated with the primary antibodies against CD147, NDUFS1, and CK18. Images were analyzed using InForm software.

Transcriptome sequencing

Transcriptome sequencing was performed by CapitalBio Technology (Beijing, China). Briefly, MIA PaCa-2 *CD147* knockdown A6 and NTC cells were cultured in a tumorsphere medium, and the tumorspheres were passaged three times to obtain stem-like cells (SPs), as previously described[23]. Then, 1×10^6 SP cells were collected and dissolved in Trizol. Sequence libraries were generated and analyzed by Solexa high-throughput sequencing technology. DESeq2 was used to analyze the differentially expressed genes (DEGs), and the DEGs were then analyzed using GO and KEGG analyses.

NAD$^+$ measurements

NAD$^+$ levels were measured using an NAD/NADH assay kit (Beyotime Biotechnology, Beijing, China). Briefly, 1×10^6 cells were lysed in 200 μL lysis buffer. One part of the lysis was heated at 60°C for 30 min, whereas another part was kept on ice. Next, 20 μL lysis without or with heated as well as 90 μL alcohol dehydrogenase working solution were transferred to a 96-well microplate, and being incubated in the dark at 37°C for 10 minutes. Then, 10 μL of the chromogenic solution were added to each well and being incubated in the dark at 37°C for 10-20 minutes. Finally, absorbance at 450 nm was measured continuously using a spectrophotometer.

Dual-luciferase reporter assay

NDUFS1 transcriptional activity was measured using the NDUFS1-luc reporter plasmid. Briefly, cells were transfected with the NDUFS1-luc reporter plasmid and the phRL-TK plasmid. At 12 h after transfection, cells were treated with 0.5 μM niclosamide. Luciferase activity was measured in the cell lysates using an Epoch™ microvolume spectrophotometer with a dual-luciferase reporter assay kit.

Methylation sequencing

Methylation sequencing was performed by the Novogene Technology Company. In brief, 1×10^6 MIA PaCa-2 adherent cells (Ads, non-CSCs) and SPs (CSCs) were collected for DNA methylation sequencing. Reduced representation bisulfite sequencing methods and the Illumina high-throughput sequencing platform were used for high-precision methylation studies of different chemical species at the reference genome level.

Methylation specific RT-PCR

Total genomic DNA was extracted and modified with bisulfite using a Bisulfite Conversion kit (Abcam). Methylation-specific primers for the *PAX2* promoter were designed using MethPrimer (**Table S5**). Bisulfite-converted genomic DNA was amplified using real-time quantitative PCR with methylation specific primers. The methylation index was calculated as the ratio of methylated DNA relative to the sum of methylated and unmethylated DNA in Ct values.

SIRT1 activity assay

The activity of SIRT1 was measured using an SIRT1 ELISA kit (Elabscience, Wuhan, CN). Briefly, 2×10^6 cells were lysed by ultrasonication. The lysate supernatant was added to each well of an ELISA plate and incubated at 37 °C for 90 min. Subsequently, 100 μL of biotinylated antibody working solution was applied per well, followed by incubation at 37 °C for 1 h. After washing, 100 μL of HRP-conjugated working solution was added and incubated at 37 °C for 30 min. Following another wash, 90 μL of TMB substrate was added, and the plate was incubated at 37 °C in the dark for 15 min. The reaction was stopped by adding 50 μL of stop solution per well. Absorbance was immediately measured at 450 nm using a microplate reader.

Chromatin immunoprecipitation (ChIP) assay

The ChIP assay was performed according to the manufacturer's protocol (SimpleChIP Plus Enzymatic Chromatin IP kit). Cells were cross linked with 1% formaldehyde for 10 min, and then washed with PBS. Crosslinking was stopped by

addition of 0.125 M glycine. Then, cells were washed in PBS, and the pellets were suspended using buffers in the ChIP Kit. The cell lysate was sonicated, the DNA was sheared to lengths of 150 to 900 bp, and the DNA was then immunoprecipitated overnight with the anti-DNMT1 antibody. Decrosslinking and processing with immunoprecipitated components were performed before real-time PCR. The quantitative PCR results were calculated as the percentage of ChIP relative to the total input ($2^{(\mathrm{Ct\ input} - \mathrm{Ct\ sample})}$).

Acetylation analysis

The immunoprecipitation (IP) assay was performed according to the manufacturer's protocol (Pierce Crosslink Immunoprecipitation Kit). Briefly, 10 μg of DNMT1 antibody were crosslinked onto Protein A/G resin. Then, 1×10^7 cells were harvested and lysed after 48 h of treatment in 1μM EX527 or SRT1720, and the lysate was incubated with antibody-crosslinked resin overnight at 4 °C. Then the antigen was subsequently eluted and detected using anti-acetyl Lysine through western blotting.

Statistical analysis

All data are presented as means ± SDs of triplicate samples from three independent experiments. The significance of differences between groups was determined using the *t*-test and one-way ANOVA with GraphPad Prism 8.0 (GraphPad Software, http://www.graphpad.com). The association between the concurrent NDUFS1 and CD147 expression and clinicopathological parameters were assessed by student *t* test and $\chi 2$ test. The Kaplan-Meier method and the log-rank test were used to

compare overall survival, defined as the time from patient surgery until death. P values were considered to be statistically significant at the level of 0.05 (*), 0.01 (**), or 0.001 (***).

Table S1. Association of the expression CD147 or NDUFS1 with patient's clinicopathological features

**Clinicopathological features**	**CD147**^{low*}	**CD147**^{high*}	***P* value**†	**NDUFS1**^{low*}	**NDUFS1**^{high*}	***P* value**†
Age (n, ≤ 60/>60 years old)	23/24	23/23	0.918	29/20	17/27	**0.048**
Gender (n, male/female)	28/20	30/16	0.492	27/23	31/13	0.102
Tumor volume (n, >35cm^3/<35 cm^3)	23/24	24/21	0.673	22/27	25/18	0.205
Tumor type (n, PDAC/ASCP and others)	11/37	6/40	0.214	39/11	38/6	0.293
Tumor grade (n, I-Ⅱ/Ⅱ/Ⅱ-Ⅲ/Ⅲ/Ⅲ-Ⅳ/Ⅳ)	6/36/5/1/0/0	4/17/14/9/1/	**0.001**	6/31/8/4/1/0	4/22/11/6/0/1	0.488
AJCC clinical grade (n, 1/2/3/4)	17/29/0/0	20/24/0/2	0.257	18/29/0/1	19/24/0/1	0.850
T stage (n, T1/T2/T3)	1/39/8	2/32/11	0.496	2/40/7	1/31/12	0.282
N stage (n, N0/N1)	22/23	28/16	0.161	22/26	28/13	**0.033**
M stage (n, M0/M1)	48/0	44/2	0.237	49/1	43/1	1.000
Metastasis (n, yes/no)	0/48	2/44	0.237	1/49	1/43	1.000
Lymph node invasion (n, yes/no)	23/21	16/26	0.187	26/21	13/26	**0.041**
Ki-67 (n, <5/5-10/10-20/20-30/>30%	5/13/10/7/9	6/13/13/7/4	0.664	5/13/13/9/6	6/13/10/5/7	0.841
P53(n, <5/5-20/20-40/40-60/60-80/>80%,)	14/5/6/5/9/5	13/3/3/5/8/1	0.573	15/6/4/8/4/9	12/2/5/2/13/7	**0.055**
OS (n, live/dead)	16/32	8/38	**0.000**	13/37	10/34	0.712

* Low (negative to weak expression), high (moderate to strong expression).

† Estimated by χ^2 test.

Table S2. The univariate Cox proportional hazard analysis of independent predictors for pancreatic cancer patient OS

Clinicopathological features (univariate analysis)	HR (95% CI)†	*P* value*
Age (n, ≤ 60/>60 years old)	1.170 (0.741-1.848)	0.500
Gender (n, male/female)	0.887 (0.550-1.432)	0.624
Tumor volume (n, >35cm^3/<35 cm^3)	1.105 (0.696-1.756)	0.671
Tumor type (n, PDAC/ASCP and others)	1.204 (0.647-2.242)	0.557
**Tumor grade (n, I-Ⅱ/Ⅱ/Ⅱ-Ⅲ/Ⅲ/Ⅲ-Ⅳ/Ⅳ)**	1.309 (1.049-1.633)	**0.017**
**AJCC clinical grade (n, 1/2/3/4)**	1.523 (1.075-2.159)	**0.018**
T stage (n, T1/T2/T3)	0.841 (0.487-1.452)	0.534
**N stage (n, N0/N1)**	1.824 (1.127-2.953)	**0.014**
M stage (n, M0/M1)	1.744 (0.426-7.141)	0.439
Metastasis (n, yes/no)	1.744 (0.426-7.141)	0.439
**Lymph node invasion (n, yes/no)**	1.896 (1.157-3.105)	**0.011**
Ki-67 (n, <5/5-10/10-20/20-30/>30%	0.915 (0.748-1.119)	0.385
P53(n, <5/5-20/20-40/40-60/60-80/>80%,)	1.093 (0.963-1.241)	0.170
**CD147/ndufs1 co-expression**	0.791 (0.651-0.960)	**0.018**
**CD147 expression (n, high/low)**	1.684 (1.084-2.705)	**0.031**
Ndufs1 expression (n, high/low)	1.319 (0.824-2.111)	0.249

†HR, hazard ratio; CI, confidence interval. *P<0.05.

Table S3. The multivariate Cox proportional hazard analysis of independent predictors for pancreatic cancer patient OS

**Clinicopathological features (multivariate analysis)**	**HR (95% CI)†**	***P* value***
Pathological grade (n, I-Ⅱ/Ⅱ/Ⅱ-III/III/III-Ⅳ/Ⅳ)	1.463 (1.118-1.915)	**0.006**
Lymph node invasion (n, yes/no)	2.878 (1.471-5.629)	**0.002**
CD147 expression (n, high/low)	1.790 (1.042-3.075)	**0.035**

†HR, hazard ratio; CI, confidence interval. *$P<0.05$.

Table S4. Human stemness gene list

RefSeq	Number Symbol	Description
NM_001265	CDX2	Caudal type homeobox 2
NM_004392	DACH1	Dachshund homolog 1 (Drosophila)
NM_178120	DLX1	Distal-less homeobox 1
NM_004405	DLX2	Distal-less homeobox 2
NM_006892	DNMT3B	DNA (cytosine-5-)-methyltransferase 3 beta
NM_004430	EGR3	Early growth response 3
NM_000125	ESR1	Estrogen receptor 1
NM_004456	EZH2	Enhancer of zeste homolog 2 (Drosophila)
NM_004496	FOXA1	Forkhead box A1
NM_021784	FOXA2	Forkhead box A2
NM_032682	FOXP1	Forkhead box P1
NM_014491	FOXP2	Forkhead box P2
NM_014009	FOXP3	Forkhead box P3
NM_002049	GATA1	GATA binding protein 1 (globin transcription factor 1)
NM_005257	GATA6	GATA binding protein 6
NM_005270	GLI2	GLI family zinc finger 2
NM_004821	HAND1	Heart and neural crest derivatives expressed 1
NM_018951	HOXA10	Homeobox A10
NM_005523	HOXA11	Homeobox A11
NM_006735	HOXA2	Homeobox A2
NM_030661	HOXA3	Homeobox A3
NM_006896	HOXA7	Homeobox A7
NM_152739	HOXA9	Homeobox A9
NM_002144	HOXB1	Homeobox B1
NM_006361	HOXB13	Homeobox B13
NM_002145	HOXB2	Homeobox B2
NM_002146	HOXB3	Homeobox B3
NM_002147	HOXB5	Homeobox B5

NM_024016	HOXB8	Homeobox B8
NM_017409	HOXC10	Homeobox C10
NM_173860	HOXC12	Homeobox C12
NM_153633	HOXC4	Homeobox C4
NM_018953	HOXC5	Homeobox C5
NM_004503	HOXC6	Homeobox C6
NM_006897	HOXC9	Homeobox C9
NM_024501	HOXD1	Homeobox D1
NM_002148	HOXD10	Homeobox D10
NM_014621	HOXD4	Homeobox D4
NM_000872	HTR7	5-hydroxytryptamine (serotonin) receptor 7 (adenylate cyclase-coupled)
NM_016358	IRX4	Iroquois homeobox 4
NM_002202	ISL1	ISL LIM homeobox 1
NM_002228	JUN	Jun proto-oncogene
NM_016270	KLF2	Kruppel-like factor 2 (lung)
NM_004235	KLF4	Kruppel-like factor 4 (gut)
NM_001004317	LIN28B	Lin-28 homolog B (C.elegans)
NM_002316	LMX1B	LIM homeobox transcription factor 1, beta
NM_002449	MSX2	Msh homeobox 2
NM_002467	MYC	V-myc myelocytomatosis viral oncogene homolog (avian)
NM_024865	NANOG	Nanog homeobox
NM_002500	NEUROD1	Neurogenic differentiation 1
NM_172390	NFATC1	Nuclear factor of activated T-cells, cytoplasmic, calcineurin-dependent 1
NM_002509	NKX2-2	NK2 homeobox 2
NM_024408	NOTCH2	Notch 2
NM_021005	NR2F2	Nuclear receptor subfamily 2, group F, member 2
NM_005806	OLIG2	Oligodendrocyte lineage transcription factor 2
NM_006192	PAX1	Paired box 1
NM_003987	PAX2	Paired box 1
NM_016734	PAX5	Paired box 5

NM_000280	PAX6	Paired box 6
NM_006194	PAX9	Paired box 9
NM_182649	PCNA	Proliferating cell nuclear antigen
NM_000325	PITX2	Paired-like homeodomain 2
NM_005029	PITX3	Paired-like homeodomain 3
NM_006237	POU4F1	POU class 4 homeobox 1
NM_004575	POU4F2	POU class 4 homeobox 2
NM_002701	POU5F1	POU class 5 homeobox 1
NM_015869	PPARG	Peroxisome proliferator-activated receptor gamma
NM_000321	RB1	Retinoblastoma 1
NM_001754	RUNX1	Runt-related transcription factor 1
NM_016932	SIX2	SIX homeobox 2
NM_005901	SMAD2	SMAD family member 2
NM_003106	SOX2	SRY (sex determining region Y)-box 2
NM_033326	SOX6	SRY (sex determining region Y)-box 6
NM_000346	SOX9	SRY (sex determining region Y)-box 9
NM_138473	SP1	Sp1 transcription factor
NM_007315	STAT1	Signal transducer and activator of transcription 1, 91kDa
NM_003150	STAT3	Signal transducer and activator of transcription 3 (acute-phase response factor)
NM_181486	TBX5	T-box 5
NM_003212	TDGF1	Teratocarcinoma-derived growth factor 1
NM_198253	TERT	Telomerase reverse transcriptase
NM_021025	TLX3	T-cell leukemia homeobox 3
NM_000376	VDR	Vitamin D (1,25-dihydroxyvitamin D3) receptor
NM_000553	WRN	Werner syndrome, RecQ helicase-like
NM_000378	WT1	Wilms tumor 1
NM_012082	ZFPM2	Zinc finger protein, multitype 2
NM_003412	ZIC1	Zic family member 1

Table S5. KEY RESOURCES

Reagent or Resource	Source	Identifier
**Antibodies**		
Anti-NDUFS1	Abcam	Ab169540
Anti-SIRT1	Abcam	Ab189494
Anti-PAX2	Abcam	Ab79389
Anti-DNMT1	Abcam	Ab188453
Anti-DNMT3A	Proteintech	20954-1-AP
Anti-DNMT3B	Proteintech	26971-1-AP
Anti-α Tubulin	Proteintech	66031-1-Ig
Goat anti-rabbit IgG	Invitrogen	31466
Goat anti-mouse IgG	Invitrogen	A16072
Anti-CD147 (HAb18IgG)	\	\
APC-conjugated anti-human CD44	BD Biosciences	559942
PE-conjugated anti-human CD133	BD Biosciences	562552
PE-conjugated anti-human CD147	BD Biosciences	130-113-
Anti-CK18	Huabio	M0407-19
Anti-5mC	Cell signaling technology	28692s
Anti-STAT3	Cell signaling technology	9139
Anti-pSTAT3^{705}	Cell signaling technology	9145
Anti-acetyl Lysine	Abcam	ab190479
**Chemicals, peptides, and recombinant proteins**		
Lipofectamine 2000 transfection reagent	Invitrogen	11668019
Oligomycin A	Selleck Chemicals	S1478
Rotenone	Selleck Chemicals	S2348
Metformin	Selleck Chemicals	S5958
EX527	Topscience	T6111
SRT1720	Topscience	T5096
2-DG	Selleck Chemicals	S4701
UK5099	Selleck Chemicals	S5317
ETO	Selleck Chemicals	S8244
BPTES	Selleck Chemicals	S7753
3-nitropropionate acid	Selleck Chemicals	S3652
N2	Gibco	17502001
B27	Gibco	A1895601
bFGF	Sigma Chemicals	GF446
EGF	Invitrogen	PHG0311
hydrocortisone	Sigma Chemicals	803146
insulin	Sigma Chemicals	11061-68-0
β-mercaptoethanol	Gibco	21985023

heparin	Stem Cell Technologies	07980
puromycin	Invitrogen	Ant-pr-1
WP1066	Selleck Chemicals	S2796
Niclsamide	Selleck Chemicals	S3030
**Critical commercial assays**		
ALDEFLUOR kit	Stem Cell Technologies	01700
ATP Assay kit	Beyotime Biotechnology	S0026
BCA Protein Assay kit	Beyotime Biotechnology	P0011
Oxygen consumption rate kit	Cayman chemical	600800
Glucose Assay kit	Jiancheng	F006-1
Lactate Assay kit	Jiancheng	A019-2
Mitochondrial Complex I Activity kit	Abcam	Ab109721
Mitochondrial Complex II Activity kit	Abcam	Ab109908
Mitochondrial Complex Ш Activity kit	Abcam	Ab124537
Mitochondrial Complex IV Activity kit	Abcam	Ab109909
Bisulfite conversion kit	Abcam	Ab117127
NAD^+/NADH assay kit	Beyotime Biotechnology	S0176S
SimpleChIP Plus Enzymatic Chromatin IP kit	Cell signaling technology	9005
Dual-luciferase reporter assay kit	promega	E1910
**Constructs**		
NDUFS1 empty vector	Genepharma	
NDUFS1 lentiviral shRNA	Genepharma	
NDUFS1 cDNA empty vector	Public plasmid library	
Human NDUFS1 lentiviral cDNA	Public plasmid library	
GV341 lentiviral expression vector	Genechem	
Human CD147 cDNA	Genechem	
Non-Target shRNA Control (pLKO.1-NTC)	Sigma-Aldrich	
CD147 pLKO.1 lentiviral shRNA (A6)	Open Biosystems	
pGIPZ empty vector	Open Biosystems	
CD147 pGIPZ lentiviral shRNA (A6)	Open Biosystems	
NDUFS1-luc reporter plasmid	Genscript	
phRL-TK plasmid	Beyotime	
**Software and Algorithms**		
Image Lab software	BIO-RAD	
FlowJo_VX	Flow Jo LLC	
Prism 8.0	GraphPad	
edgeR	Biorender	
Limma	Biorender	
InForm	Vectra	

Primer sequences

SOX2	HUADA	Forward	5'-TGGACAGTTACGCGCACAT-3'
		Reverse	5'-CGAGTAGGACATGCTGTAGGT-3'
NANOG	HUADA	Forward	5'-CCCCAGCCTTTACTCTTCCTA-3'
		Reverse	5'-CCAGGTTGAATTGTTCCAGGTC-3'
KLF4	HUADA	Forward	5'-CCCACATGAAGCGACTTCCC-3'
		Reverse	5'-CAGGTCCAGGAGATCGTTGAA-3'
OCT4	HUADA	Forward	5'-GGGCTCTCCCATGCATTCAAAC-3'
		Reverse	5'-ACCTTCCCTCCAACCAGTTGC-3'
NDUFS1	HUADA	Forward	5'-TTAGCAAATCACCCATTGGACTG-3'
		Reverse	5'-CCCCTCTAAAAATCGGCTCCTA-3'
CD147	HUADA	Forward	5'-ACTCCTCACCTGCTCCTTGA-3'
		Reverse	5'-GCCTCCATGTTCAGGTTCTC-3'
SIRT1	HUADA	Forward	5'-TAGCCTTGTCAGATAAGGAAGGA-3'
		Reverse	5'-ACAGCTTCACAGTCAACTTTGT-3'
PAX2	HUADA	Forward	5'-TGGTCGGGTTCTGTCGTTTGTATT-3'
		Reverse	5'-TCAGCAAAATCCTGGGCAG-3'
HOXB2	HUADA	Forward	5'-CGCCAGGATTCACCTTTCCTT-3'
		Reverse	5'-CCCTGTAGGCTAGGGGAGAG-3'
18sRNA	HUADA	Forward	5'-CAGCCACCCGAGATTGAGCA-3'
		Reverse	5'-TAGTAGCGACGGGCGGTGTG-3'
Methylation Specific Primers for PAX2 promoters	Tsingke Bio	Forward	5'-GGGCCGCACACCCTGGTG-3'
		Reverse	5'-GTGTGAACCAGCTCGGGGG-3'

siRNA sequences

NDUFS1	Genepharma	siRNA-1:	5'-UAAGCAAAUCUUUGAGAGCTT-3'
		siRNA-2:	5'-UUGGAACGCAAAUCUGUGCTT-3'
		siRNA-3:	5'-UAGCAUUAAACAGUGGUGCTT-3'
SIRT1	Genepharma	siRNA-1:	5'-UAAGCAAAUCUUUGAGAGCTT-3'
		siRNA-2:	5'-UUGGAACGCAAAUCUGUGCTT-3'
		siRNA-3:	5'-UAGCAUUAAACAGUGGUGCTT-3'
DNMT1	Genepharma	siRNA:	5'-GCACCUCAUUUGCCGAAUATT-3'
DNMT3A	Genepharma	siRNA:	5'-CGAGGUCAAACUCCAUAAATT-3'
DNMT3B	Genepharma	siRNA:	5'-GUACCAUGCUCUGGAGAAATT-3'
PAX2	Genepharma	siRNA:	5'-CCAGCGUCUCUUCCAUCAATT-3'

Table S6. Characteristics of pancreatic cancer patients

Clinical features	Groups	patients (%)
**Age, year**	Mean±SD	61.9±10.9
**Gender**	Male	63 (63.6)
	Female	36 (36.4)
**Tumor volume**	≥35cm^2	48 (49.5)
	＜35cm^2	49 (50.5)
**Tumor type**	PDAC	82 (82.8)
	ASCP and others	17 (17.2)
**Tumor grade**	I -II	10 (10.1)
	II / II-III	56 (76.8)
	III / Ⅲ-Ⅳ	12 (12.1)
	Ⅳ	1 (1)
**AJCC clinical grade**	1	37 (38.5)
	2	57 (59.4)
	3	0 (0)
	4	2 (2.1)
**T stage**	T1	3 (3.1)
	T2	75 (76.55)
	T3	20 (20.4)
**N stage**	N0	50 (53.8)
	N1	43 (46.2)
**M stage**	M0	97 (98)
	M1	2 (2)
**Tumor metastasis**	Yes	2 (2)
	No	97 (98)
**Lymph node invasion**	Yes	43 (47.8)
	No	47 (52.2)
**Ki-67**	<5%	11 (12)
	≥5%, <10%	28 (30.4)
	≥10%, <20%	25 (27.2)
	≥20%, <30%	14 (15.2)
	≥30%	14 (15.2)
**P53**	<5%	27 (29.3)
	⩾5%,<20%	8 (8.7)
	⩾20%,<40%	10 (10.9)
	⩾40%,<60%	11 (12)
	⩾60%,<80%	19 (20.7)
	⩾80%	17 (18.5)
**OS (overall survival)**	Mean (range)	10 (6-38)
	Live	25 (25.3)
	Dead	74 (74.7)

Figure. S1

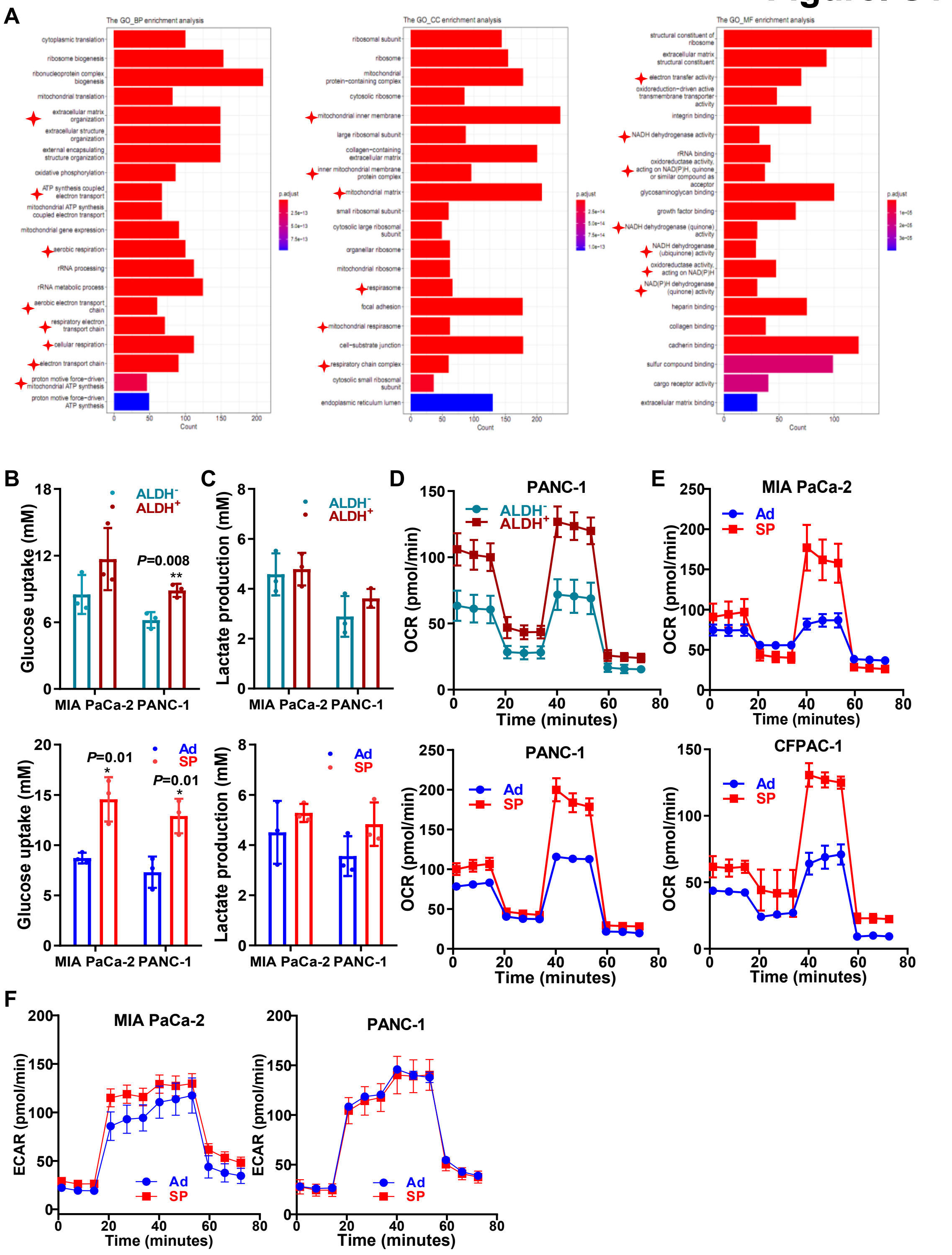

A
The GO_BP enrichment analysis
The GO_CC enrichment analysis
The GO_MF enrichment analysis
B
C
D
E
F
Glucose uptake (mM)
Lactate production (mM)
OCR (pmol/min)
ECAR (pmol/min)
Time (minutes)
MIA PaCa-2 PANC-1
ALDH-
ALDH+
Ad
SP
P=0.008
P=0.01
PANC-1
MIA PaCa-2
CFPAC-1

Figure. S2

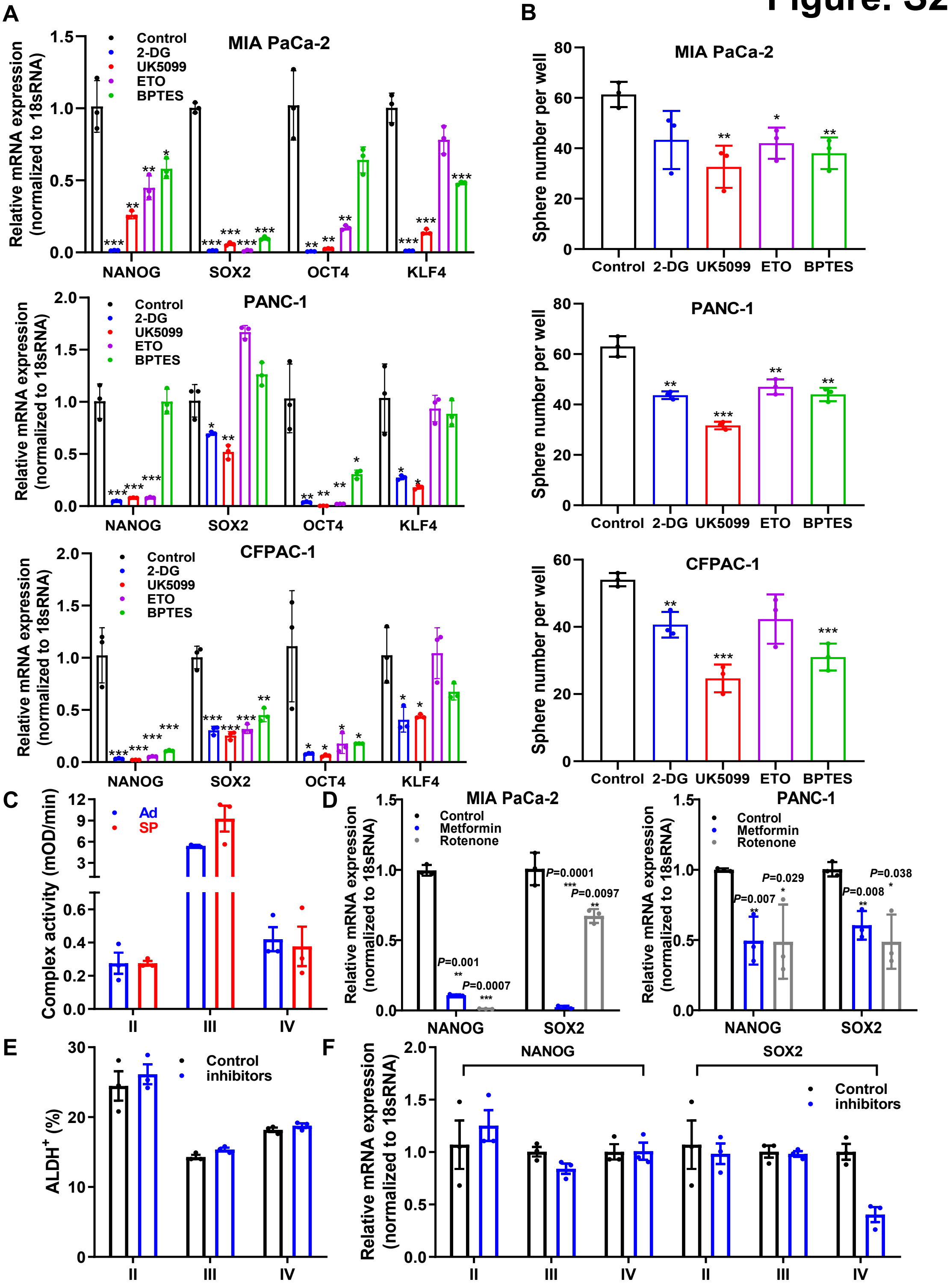
A
B
C
D
E
F
MIA PaCa-2
PANC-1
CFPAC-1
Relative mRNA expression (normalized to 18sRNA)
Sphere number per well
Control
2-DG
UK5099
ETO
BPTES
NANOG
SOX2
OCT4
KLF4
Complex activity (mOD/min)
Ad
SP
II
III
IV
Metformin
Rotenone
P=0.001
P=0.0007
P=0.0001
P=0.0097
P=0.007
P=0.029
P=0.008
P=0.038
ALDH+ (%)
inhibitors

Figure. S3

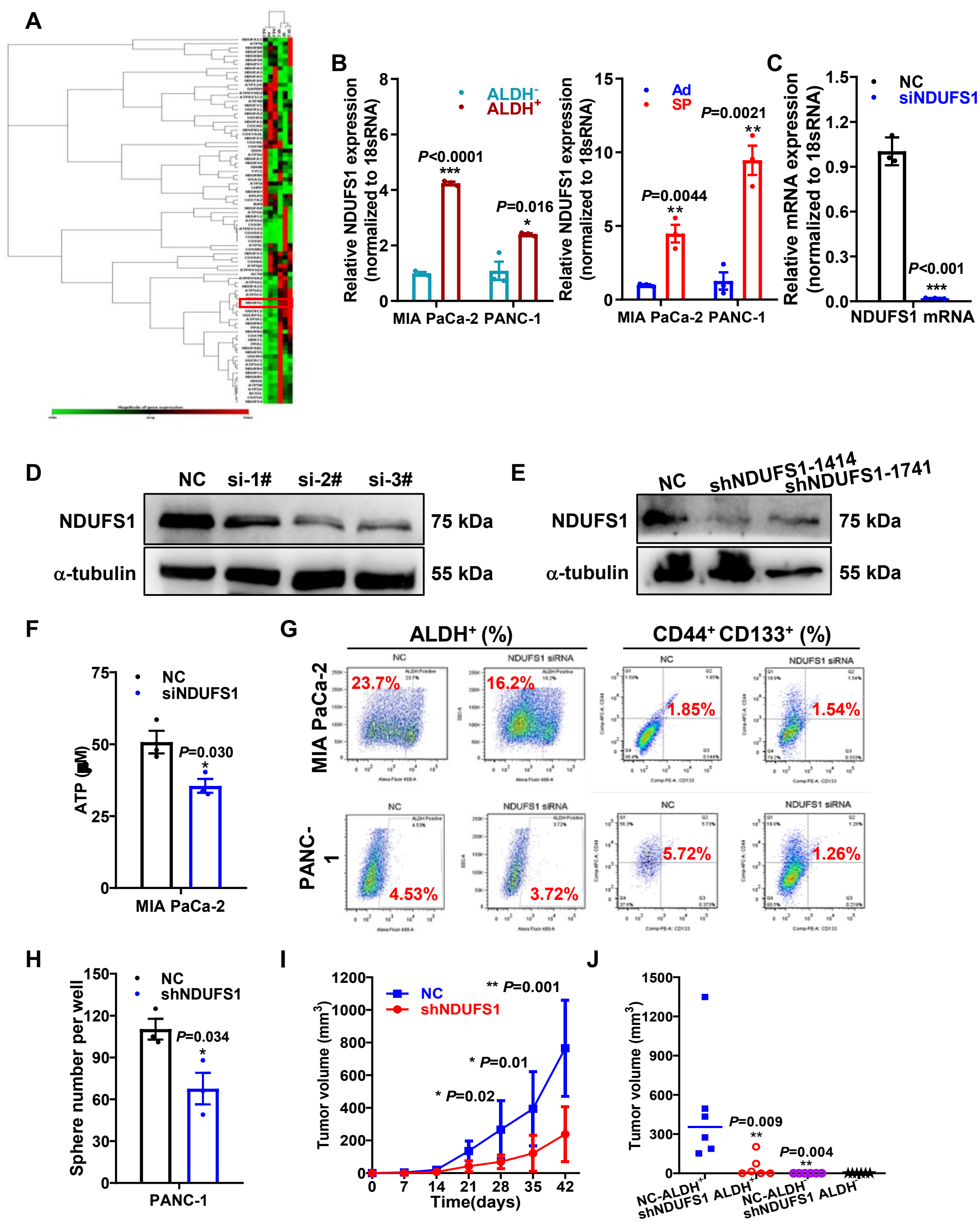
A
B
Relative NDUFS1 expression (normalized to 18sRNA)
ALDH⁻
ALDH⁺
P<0.0001
***
P=0.016
*
MIA PaCa-2
PANC-1
Ad
SP
P=0.0044
**
P=0.0021
**
C
Relative mRNA expression (normalized to 18sRNA)
NC
siNDUFS1
P<0.001
***
NDUFS1 mRNA
D
NC
si-1#
si-2#
si-3#
NDUFS1
α-tubulin
75 kDa
55 kDa
E
NC
shNDUFS1-1414
shNDUFS1-1741
NDUFS1
α-tubulin
75 kDa
55 kDa
F
ATP (μM)
NC
siNDUFS1
P=0.030
*
MIA PaCa-2
G
ALDH⁺ (%)
CD44⁺ CD133⁺ (%)
MIA PaCa-2
PANC-1
NC
NDUFS1 siRNA
23.7%
16.2%
1.85%
1.54%
4.53%
3.72%
5.72%
1.26%
H
Sphere number per well
NC
shNDUFS1
P=0.034
*
PANC-1
I
Tumor volume (mm³)
NC
shNDUFS1
** P=0.001
* P=0.01
* P=0.02
Time(days)
J
Tumor volume (mm³)
P=0.009
**
P=0.004
**
NC-ALDH⁺
shNDUFS1 ALDH⁺
NC-ALDH⁻
shNDUFS1 ALDH⁻

Figure. S4

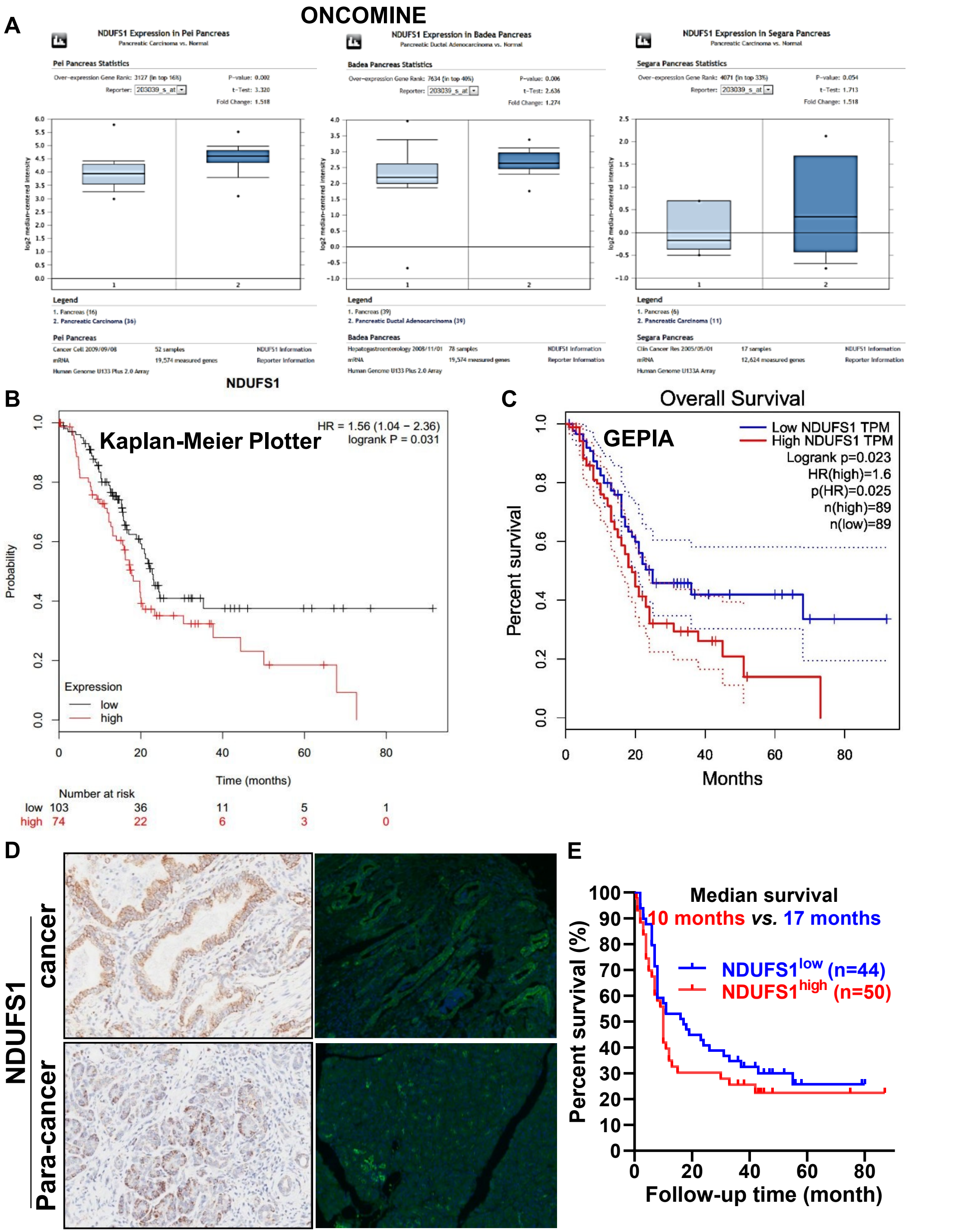

A
ONCOMINE
NDUFS1 Expression in Pei Pancreas
Pancreatic Carcinoma vs. Normal
Pei Pancreas Statistics
Over-expression Gene Rank: 3127 (in top 16%)
P-value: 0.002
t-Test: 3.320
Fold Change: 1.518
Reporter: 203039_s_at
log2 median-centered intensity
Legend
1. Pancreas (16)
2. Pancreatic Carcinoma (36)
Pei Pancreas
Cancer Cell 2009/09/08
52 samples
NDUFS1 Information
mRNA
19,574 measured genes
Reporter Information
Human Genome U133 Plus 2.0 Array
NDUFS1 Expression in Badea Pancreas
Pancreatic Ductal Adenocarcinoma vs. Normal
Badea Pancreas Statistics
Over-expression Gene Rank: 7634 (in top 40%)
P-value: 0.006
t-Test: 2.636
Fold Change: 1.274
Reporter: 203039_s_at
Legend
1. Pancreas (39)
2. Pancreatic Ductal Adenocarcinoma (39)
Badea Pancreas
Hepatogastroenterology 2008/11/01
78 samples
NDUFS1 Information
mRNA
19,574 measured genes
Reporter Information
Human Genome U133 Plus 2.0 Array
NDUFS1 Expression in Segara Pancreas
Pancreatic Carcinoma vs. Normal
Segara Pancreas Statistics
Over-expression Gene Rank: 4071 (in top 33%)
P-value: 0.054
t-Test: 1.713
Fold Change: 1.518
Reporter: 203039_s_at
Legend
1. Pancreas (6)
2. Pancreatic Carcinoma (11)
Segara Pancreas
Clin Cancer Res 2005/05/01
17 samples
NDUFS1 Information
mRNA
12,624 measured genes
Reporter Information
Human Genome U133A Array
B
NDUFS1
Kaplan-Meier Plotter
HR = 1.56 (1.04 − 2.36)
logrank P = 0.031
Probability
Time (months)
Expression
low
high
Number at risk
low 103 36 11 5 1
high 74 22 6 3 0
C
Overall Survival
GEPIA
Low NDUFS1 TPM
High NDUFS1 TPM
Logrank p=0.023
HR(high)=1.6
p(HR)=0.025
n(high)=89
n(low)=89
Percent survival
Months
D
NDUFS1
cancer
Para-cancer
E
Median survival
10 months vs. 17 months
NDUFS1 low (n=44)
NDUFS1 high (n=50)
Percent survival (%)
Follow-up time (month)

Figure. S5

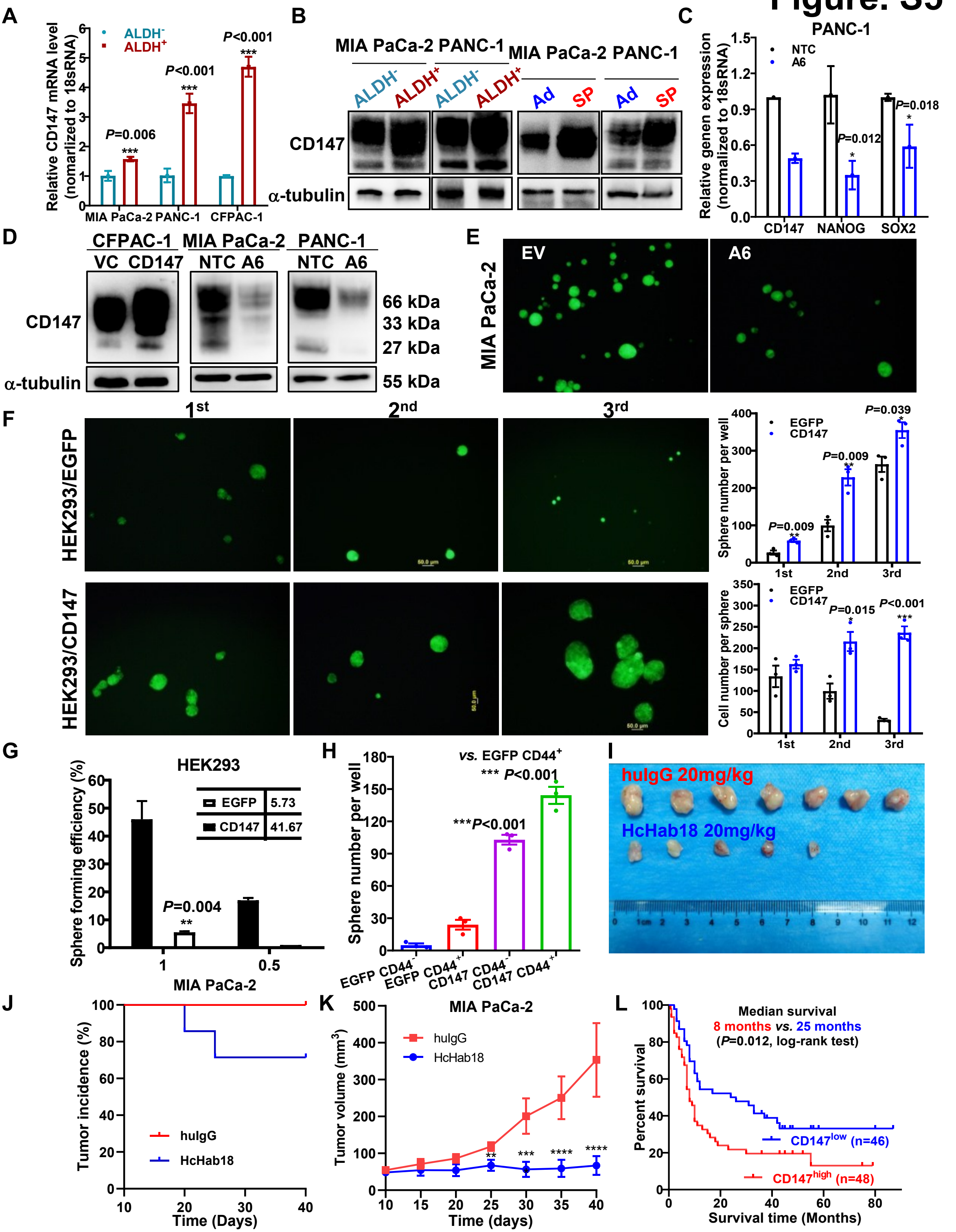
A
ALDH-
ALDH+
Relative CD147 mRNA level (nomarlized to 18sRNA)
P=0.006
P<0.001
P<0.001
MIA PaCa-2 PANC-1 CFPAC-1
B
MIA PaCa-2 PANC-1
MIA PaCa-2 PANC-1
ALDH- ALDH+ ALDH- ALDH+
Ad SP Ad SP
CD147
α-tubulin
C
PANC-1
NTC
A6
Relative genen expression (normalized to 18sRNA)
P=0.012
P=0.018
CD147 NANOG SOX2
D
CFPAC-1 MIA PaCa-2 PANC-1
VC CD147 NTC A6 NTC A6
CD147
α-tubulin
66 kDa
33 kDa
27 kDa
55 kDa
E
MIA PaCa-2
EV
A6
F
1st
2nd
3rd
HEK293/EGFP
HEK293/CD147
EGFP
CD147
Sphere number per well
P=0.009
P=0.009
P=0.039
1st 2nd 3rd
Cell number per sphere
P=0.015
P<0.001
1st 2nd 3rd
G
HEK293
EGFP 5.73
CD147 41.67
Sphere forming efficiency (%)
P=0.004
1 0.5
MIA PaCa-2
H
vs. EGFP CD44+
*** P<0.001
***P<0.001
Sphere number per well
EGFP CD44-
EGFP CD44+
CD147 CD44-
CD147 CD44+
I
huIgG 20mg/kg
HcHab18 20mg/kg
J
Tumor incidence (%)
huIgG
HcHab18
Time (Days)
K
MIA PaCa-2
huIgG
HcHab18
Tumor volume (mm3)
Time (days)
L
Median survival
8 months vs. 25 months
(P=0.012, log-rank test)
Percent survival
CD147low (n=46)
CD147high (n=48)
Survival time (Months)

Figure. S6

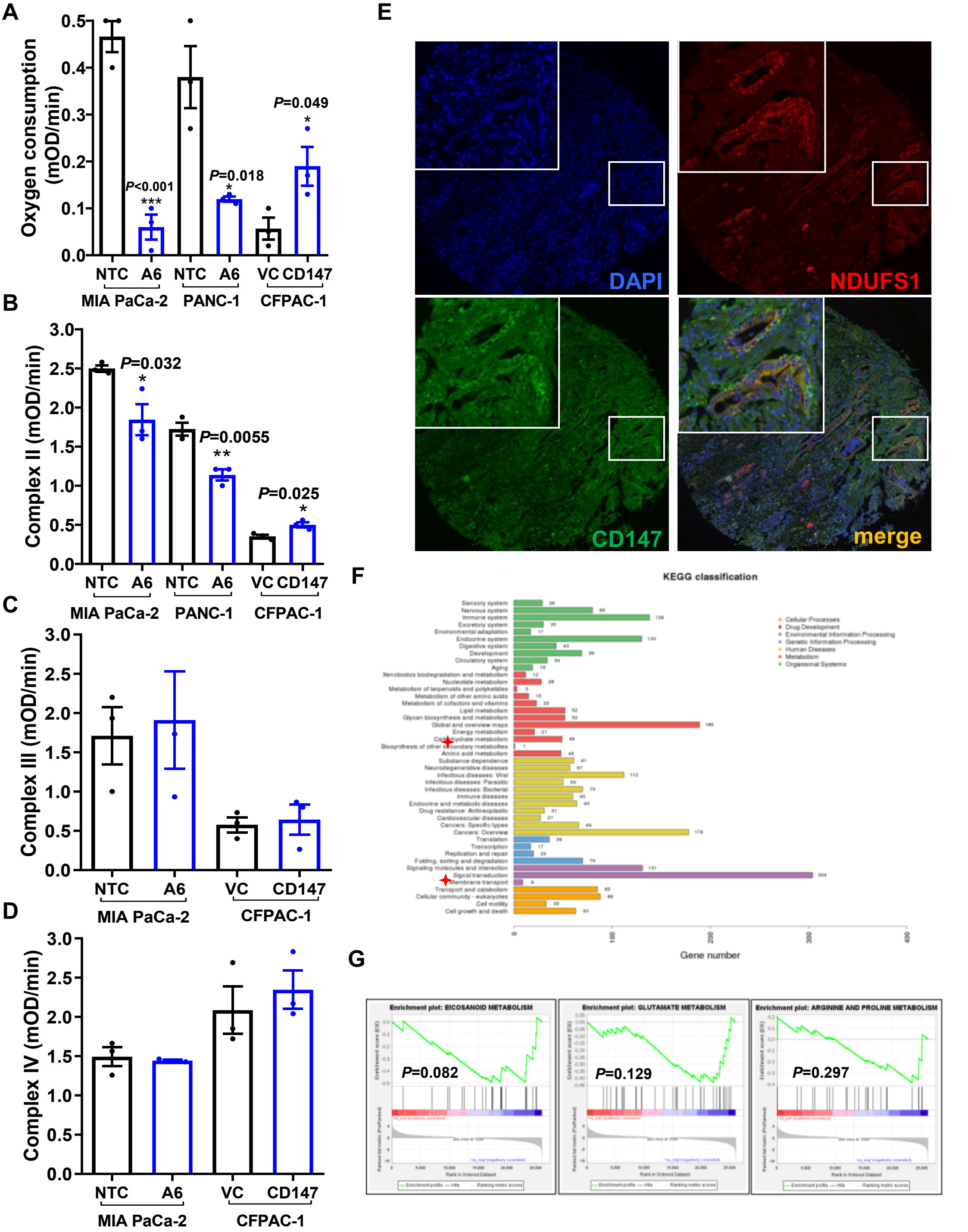

A
Oxygen consumption (mOD/min)
P<0.001
***
P=0.018
*
P=0.049
*
NTC A6 NTC A6 VC CD147
MIA PaCa-2 PANC-1 CFPAC-1
B
Complex II (mOD/min)
P=0.032
*
P=0.0055
**
P=0.025
*
NTC A6 NTC A6 VC CD147
MIA PaCa-2 PANC-1 CFPAC-1
C
Complex III (mOD/min)
NTC A6 VC CD147
MIA PaCa-2 CFPAC-1
D
Complex IV (mOD/min)
NTC A6 VC CD147
MIA PaCa-2 CFPAC-1
E
DAPI
NDUFS1
CD147
merge
F
KEGG classification
Gene number
G
Enrichment plot: EICOSANOID METABOLISM
P=0.082
Enrichment plot: GLUTAMATE METABOLISM
P=0.129
Enrichment plot: ARGININE AND PROLINE METABOLISM
P=0.297

Figure. S7

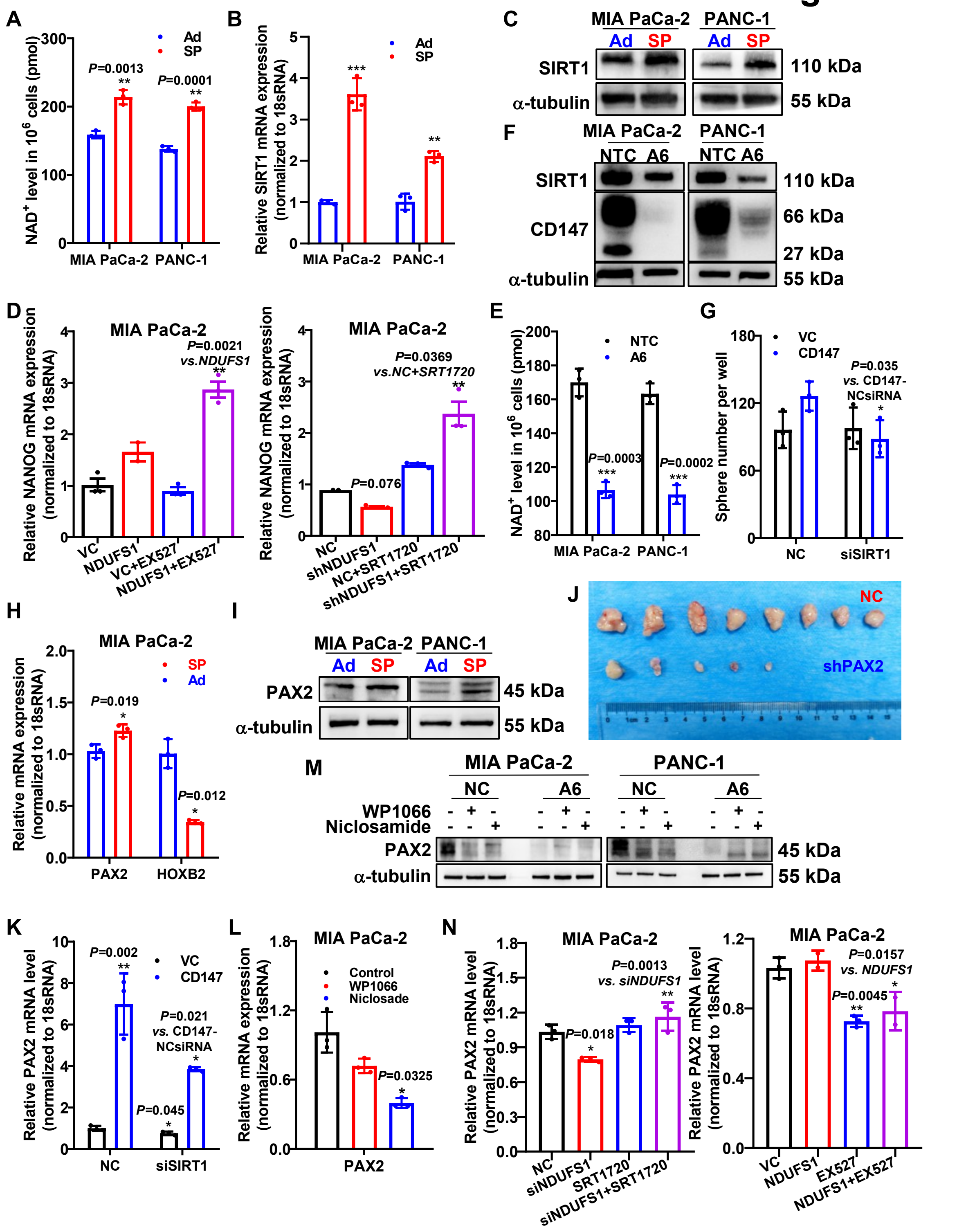
A
NAD+ level in 10^6 cells (pmol)
Ad
SP
P=0.0013
P=0.0001
MIA PaCa-2 PANC-1
B
Relative SIRT1 mRNA expression (normalized to 18sRNA)
C
MIA PaCa-2 PANC-1
SIRT1
α-tubulin
110 kDa
55 kDa
F
NTC A6
CD147
66 kDa
27 kDa
D
MIA PaCa-2
Relative NANOG mRNA expression (normalized to 18sRNA)
P=0.0021 vs.NDUFS1
VC NDUFS1 VC+EX527 NDUFS1+EX527
P=0.0369 vs.NC+SRT1720
P=0.076
NC shNDUFS1 NC+SRT1720 shNDUFS1+SRT1720
E
NTC
A6
P=0.0003
P=0.0002
G
Sphere number per well
VC
CD147
P=0.035 vs. CD147-NCsiRNA
NC siSIRT1
H
Relative mRNA expression (normalized to 18sRNA)
P=0.019
P=0.012
PAX2 HOXB2
I
PAX2
45 kDa
J
NC
shPAX2
M
WP1066
Niclosamide
K
Relative PAX2 mRNA level (normalized to 18sRNA)
P=0.002
P=0.021 vs. CD147-NCsiRNA
P=0.045
L
Control
WP1066
Niclosamide
P=0.0325
N
P=0.0013 vs. siNDUFS1
P=0.018
NC siNDUFS1 SRT1720 siNDUFS1+SRT1720
P=0.0157 vs. NDUFS1
P=0.0045
VC NDUFS1 EX527 NDUFS1+EX527

Figure. S8

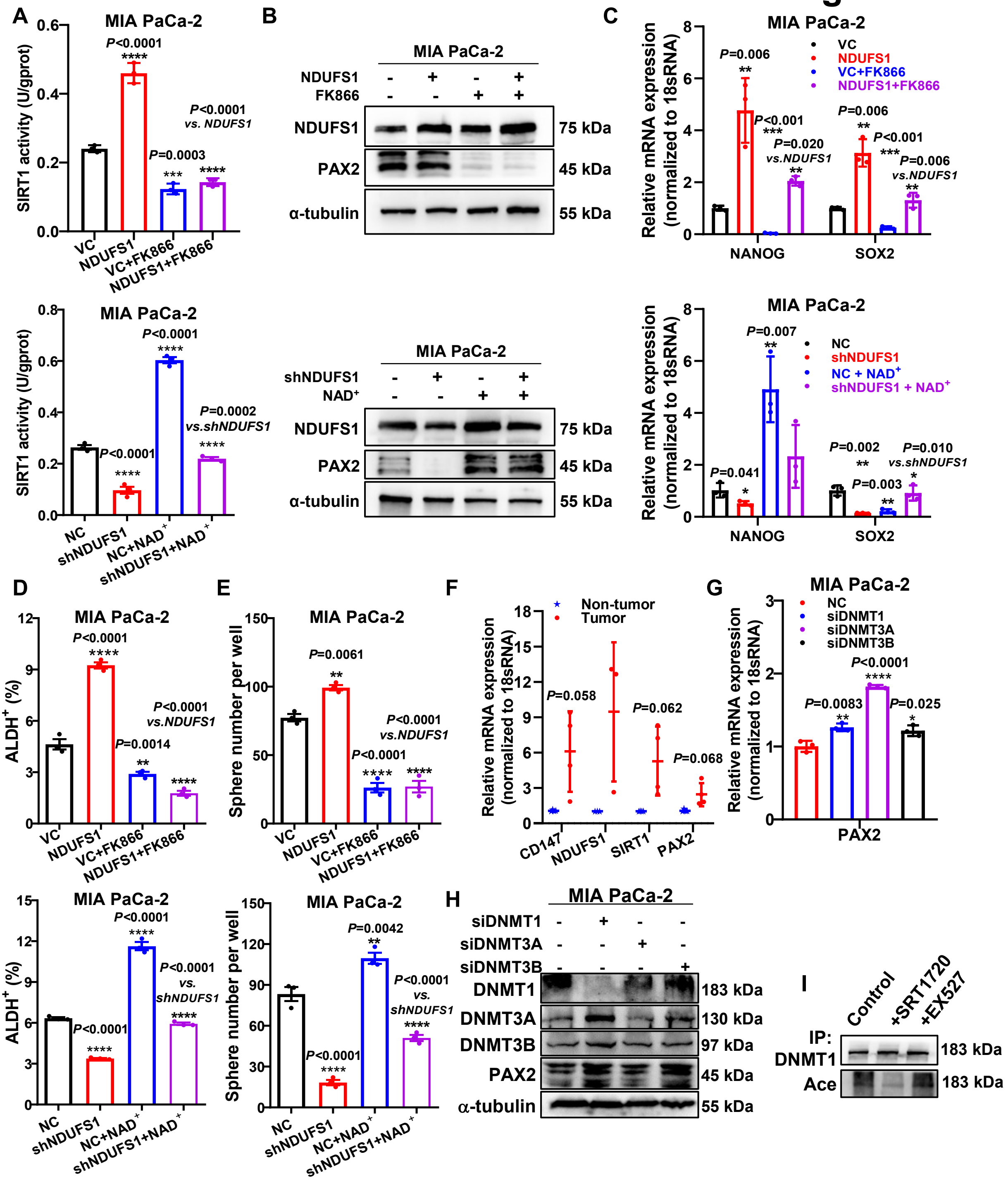

A
MIA PaCa-2
SIRT1 activity (U/gprot)
VC
NDUFS1
VC+FK866
NDUFS1+FK866
NC
shNDUFS1
NC+NAD+
shNDUFS1+NAD+
B
MIA PaCa-2
NDUFS1
FK866
PAX2
α-tubulin
75 kDa
45 kDa
55 kDa
shNDUFS1
NAD+
C
Relative mRNA expression
(normalized to 18sRNA)
NANOG
SOX2
D
ALDH+ (%)
E
Sphere number per well
F
Non-tumor
Tumor
CD147
SIRT1
PAX2
G
NC
siDNMT1
siDNMT3A
siDNMT3B
H
DNMT1
DNMT3A
DNMT3B
183 kDa
130 kDa
97 kDa
I
Control
+SRT1720
+EX527
IP:
DNMT1
Ace

Supplementary Figure Legends

Figure S1. Metabolism signatures in pancreatic CSCs.

(A) GO enrichment analysis of DEGs between patients with high- or low- mRNAsi scores. *Left*, BP, Biological Process; *Middle*: CC, Cellular Component; *Right*: MF, Molecular Function. **(B-C)** Glucose uptake (B) and lactate production (C) in CSCs and non-CSCs obtained from $ALDH^+/ALDH^-$ cells (*up*) and SP/Ad (*down*). **(D-F)** Rates of oxygen consumption (OCR, D-E) and extracellular acidification (ECAR, F) in CSCs and non-CSCs obtained from $ALDH^+/ALDH^-$ cells and SP/Ad.

Figure S2. Complex I-driven OXPHOS sustains pancreatic CSC stemness.

(A-B) *NANOG*, *SOX2, OCT4,* and *KLF4* mRNA levels (A) and tumorsphere numbers (B) in MIA PaCa-2 (*upper*), PANC-1 (*middle*) and CFPAC-1 (*bottom*) cells without (Control) or with 2-DG (1 mM), UK5099 (50 µM), ETO (50 µM), or BPTES (10 µM) treatment for 24 hours. **(C)** Activities of the mitochondrial complexes II/III/IV in SP/Ad. **(D)** *NANOG* and *SOX2* mRNA levels in MIA PaCa-2 (*left*) and PANC-1 (*right*) cells treated without (Control) or with Rotenone. Metformin was used as a positive control. **(E-F)** $ALDH^+$ subpopulation analysis (E) and *NANOG* and *SOX*2 mRNA levels (F) in MIA PaCa-2 cells treated without (Control) or with inhibitors of complex II (3-nitropropanoic acid, 3 mM), complex III (antimycin A, 1 µM), and complex IV (NaN3, 1 mM) for 24 hours.

Figure S3. NDUFS1 maintains pancreatic CSC stemness.

(**A**) Heatmap of DEGs between SP and Ad of MIA PaCa-2 cells, analyzed using the Human Mitochondrial Energy Metabolism RT^2 Profiler PCR Array. (**B**) NDUFS1 mRNA levels in ALDH$^+$ or ALDH$^-$ subpopulations, as well as in SP or Ad. (**C-E**) Levels of NDUFS1 mRNA (C) and protein (D-E) in MIA PaCa-2 cells either without or with siRNA or shRNA interference. (**F**) ATP production in MIA PaCa-2 siNDUFS1 cells and their corresponding negative control (NC) cells. (**G**) ALDH$^+$ (*left*) and CD44$^+$CD133$^+$ (*right*) subpopulation analysis in MIA PaCa-2 and PANC-1 siNDUFS1 cells and their respective NC cells. (**H**) Tumorsphere numbers in PANC-1 shNDUFS1 cells and their NC counterparts. (**I-J**) Tumor volume in nude mice injected with 1×10^6 PANC-1 cells (I) and in NOD-SCID mice injected with 50,000 ALDH$^+$ or ALDH$^-$ MIA PaCa-2 cells (J), under conditions without or with *NDUFS1* knock-down.

Figure S4. High NDUFS1 expression correlates with poor clinical outcomes in pancreatic cancer patients.

(**A**) *NDUFS*1 mRNA levels in pancreatic cancer tissues and normal pancreatic tissues sourced from the Oncomine database. (**B-C**) Kaplan-Meier Plotter and GEPIA were employed for survival analysis of pancreatic cancer patients with either high- or low- *NDUFS1* mRNA expression. (**D-E**) Tissue immunofluorescence staining (D) and survival analysis (E) of pancreatic cancer patients with high- or low- NDUFS1 protein expression.

Figure S5. CD147 promotes the CSC features of pancreatic cancer cells.

(**A**) *CD147* mRNA levels in $ALDH^+$ and $ALDH^-$ subpopulations. (**B**) CD147 protein levels in CSCs and non-CSCs from $ALDH^+$/$ALDH^-$ subpopulations as well as SP/Ad. (**C**) *CD147*, *NANOG*, and *SOX2* mRNA levels in PANC-1 A6 or non-target control (NTC) cells. (**D**) CD147 protein levels in A6 or NTC cells from MIA PaCa-2 and PANC-1 cells, as well as in CD147 or vector control (VC) cells from CFPAC-1 cells. (**E**) Fluorescence images of tumorspheres in 6×10^3 MIA PaCa-2 cells transfected with CD147 pGIPZ shRNA A6 or empty vector (EV). (**F**) Tumorsphere numbers and the number of cells per tumorsphere were quantified after different passages of 6×10^3 HEK293 CD147/EGFP cells. (**G**) Efficiency of tumorsphere formation in HEK293 CD147/EGFP cells when 0.5 and 1 cell per well were inoculated in 96-well ultra-low attachment plates. (**H**) Tumorsphere numbers in 500 cells from the $CD44^+$ and $CD44^-$ subpopulations of HEK293 CD147/EGFP cells. (**I-K**) Tumor images (I), tumor incidence (J) and tumor growth curve (K) in MIA PaCa-2 xenograft-bearing nude mice treated with either anti-CD147 human HcHab18 or control human IgG (20 mg/kg, intravenous injection twice weekly). (**L**) Survival analysis of pancreatic cancer patients with high- or low- expression of CD147.

Figure S6. CD147 promotes OXPHOS of pancreatic cancer cells.

(**A**) Oxygen consumption rate (Cayman Oxygen consumption rate assay Kit) in MIA PaCa-2 and PANC-1 A6/NTC cells as well as in CFPAC-1 CD147/VC cells. (**B-D**) Activities of the mitochondrial complexes II (B), III (C) and IV (D) in MIA PaCa-2

and PANC-1 A6/NTC cells as well as in CFPAC CD147/VC cells. (**E**) Representative immunostaining images showing the co-expression of CD147 and NDUFS1 in pancreatic cancer tissues. (**F**) KEGG classification of DEGs from transcriptome sequencing of MIA PaCa-2 A6 SP and NTC SP. (**G**) Metabolic pathway GSEA analysis of DEGs from transcriptome sequencing of MIA PaCa-2 A6 SP and NTC SP.

Figure S7. CD147-NDUFS1 signaling promotes pancreatic CSC stemness *via* SIRT1-mediated *PAX2* hypomethylation.

(**A**) NAD^+ contents in MIA PaCa-2 and PANC-1 SP/Ad cells. (**B-C**) *SIRT1* mRNA (B) and protein (C) levels in MIA PaCa-2 and PANC-1 SP/Ad cells. (**D**) *NANOG* mRNA levels in NDUFS1 knocked-in (NDUFS1) or VC cells treated without or with 1 μM EX527, and in *NDUFS1* knockdown (shNDUFS1) or NC cells treated without or with 1 μM SRT1720. (**E-F**) NAD^+ contents (E) and CD147/SIRT1 protein levels (F) in MIA PaCa-2 and PANC-1 A6/NTC cells. (**G**) Tumorsphere numbers in CD147/VC cells without or with *SIRT1* interference. (**H**) *HOXB2* and *PAX2* mRNA levels in MIA PaCa-2 SP/Ad cells. (**I**) *PAX2* protein levels in MIA PaCa-2 and PANC-1 SP/Ad cells. (**J**) Tumor images in nude mice injected with 1×10^6 MIA PaCa-2 cells without (NC) or with *PAX2* knockdown (shPAX2). (**K**) *PAX2* mRNA levels in CD147/VC cells with or without *SIRT1* interference. (**L**) *PAX2* mRNA levels in MIA PaCa-2 cells treated without or with 1 μM WP1066 or 0.5 μM niclosamide. (**M**) Protein levels of PAX2 in NC/A6 cells with or without 1 μM WP1066 or 0.5 μM niclosamide for 24 hours. (**N**) *PAX2* mRNA levels in MIA PaCa-2 siNDUFS1/NC cells treated without

or with 1 μM SRT1720 (*left*), and in MIA PaCa-2 NDUFS1/VC cells treated without or with1 μM EX527 (*right*).

Figure S8. CD147-NDUFS1 signaling promotes pancreatic CSC stemness *via* SIRT1/DNMT1-mediated *PAX2* hypomethylation.

(A-E) SIRT1 activity (A)**,** *PAX2* protein levels (B), NANOG and SOX2 mRNA levels (C), $ALDH^+$ subpopulations (D) and tumorsphere numbers (E) in MIA PaCa-2 NDUFS1/VC cells treated without or with 0.01 μM FK866 or MIA PaCa-2 shNDUFS1/NC cells treated without or with 1 mM NAD^+. **(F)** *CD147, NDUFS1, SIRT1 and PAX2* mRNA levels in paired pancreatic cancer and normal tissues (n=4). **(G-H)** *PAX2* mRNA (G) and protein (H) levels of in MIA PaCa-2 cells without or with siDNMT1, siDNMT3A, or siDNMT3B interference. **(I)** Acetylation levels of DNMT1 in MIA PaCa-2 cells treated with 1 μM EX527, 1 μM SRT1720, or solvent (control).
